# Fatty acid transport protein 4 is required for incorporation of saturated ultralong-chain fatty acids into epidermal ceramides and monoacylglycerols

**DOI:** 10.1038/s41598-019-49684-y

**Published:** 2019-09-13

**Authors:** Meei-Hua Lin, Fong-Fu Hsu, Debra Crumrine, Jason Meyer, Peter M. Elias, Jeffrey H. Miner

**Affiliations:** 1 Division of Nephrology, Washington University School of Medicine, 4523 Clayton Ave., St. Louis, MO 63110 United States; 2 Division of Endocrinology, Metabolism, and Lipid Research, Department of Medicine, Washington University School of Medicine, 4523 Clayton Ave., St. Louis, MO 63110 United States; 3 Dermatology Service, VA Medical Center and Department of Dermatology, University of California-San Francisco, 4150 Clement St., San Francisco, CA 94121 United States; 4 Department of Cell Biology and Physiology, Washington University School of Medicine, 4523 Clayton Ave., St. Louis, MO 63110 United States

**Keywords:** Skin diseases, Fatty acids, Sphingolipids, Mass spectrometry, Lipidomics

## Abstract

Fatty acid transport protein 4 (FATP4) is an acyl-CoA synthetase that is required for normal permeability barrier in mammalian skin. FATP4 (*SLC27A4*) mutations cause ichthyosis prematurity syndrome, a nonlethal disorder. In contrast, *Fatp4*^−/−^ mice die neonatally from a defective barrier. Here we used electron microscopy and lipidomics to characterize defects in *Fatp4*^−/−^ mice. Mutants showed lamellar body, corneocyte lipid envelope, and cornified envelope abnormalities. Lipidomics identified two lipids previously speculated to be present in mouse epidermis, sphingosine β-hydroxyceramide and monoacylglycerol; mutants displayed decreased proportions of these and the two ceramide classes that carry ultralong-chain, amide-linked fatty acids (FAs) thought to be critical for barrier function, unbound ω-O-acylceramide and bound ω-hydroxyceramide, the latter constituting the major component of the corneocyte lipid envelope. Other abnormalities included elevated amounts of sphingosine α-hydroxyceramide, phytosphingosine non-hydroxyceramide, and 1-O-acylceramide. Acyl chain length alterations in ceramides also suggested roles for FATP4 in esterifying saturated non-hydroxy and β-hydroxy FAs with at least 25 carbons and saturated or unsaturated ω-hydroxy FAs with at least 30 carbons to CoA. Our lipidomic analysis is the most thorough such study of the *Fatp4*^−/−^ mouse skin barrier to date, providing information about how FATP4 can contribute to barrier function by regulating fatty acyl moieties in various barrier lipids.

## Introduction

Mammalian skin defends against biological, chemical, and mechanical assaults and acts as a barrier to prevent water loss. Its permeability barrier is established by a series of differentiation events to form the outermost, cornified layer with three components. (1) The cornified envelope of dead, flattened corneocytes^1^ is a tough, water-insoluble protein sac that envelops keratin fibers. (2) Intercellular lipid lamellae form from secreted and processed lipids from lamellar bodies in granulocytes^2^. (3) The corneocyte lipid envelope is covalently linked to involucrin in the cornified envelope, functioning as a scaffold for organizing intercellular lipid lamellae, for intercorneocyte cohesion, or as a semipermeable membrane^3^.

The lipid lamellae are composed of three major types of lipids—free fatty acid (FFA), ceramide, and cholesterol—at an approximately equimolar ratio^4^. Ceramide is composed of a fatty acid (FA) and a sphingosine as the long-chain base. With many possible modifications, ceramides are a large family of lipids^5^. Epidermal FAs can contribute to formation of complex lipids or be present in a free form. Several proteins have been implicated in facilitating the uptake of long-chain FAs (LCFA; C12-C20) in mammalian cells^6^, including a family of fatty acid transport proteins (FATPs), also known as very-long-chain acyl-CoA synthetases^7,8^. Clinical findings and animal model studies suggest important roles for these candidate transporters in the skin^6^.

The FATP family comprises six membrane proteins that mediate uptake of LCFA and very-long-chain FAs (VLCFA; ≥ C22)^9–12^. FATPs show diverse subcellular localizations, including plasma and organellar membranes^13,14^. FATPs exhibit acyl-CoA synthetase activity and are implicated in facilitating uptake of FAs by vectorial acylation^15^. The deposition of FATPs in the skin varies substantially^16^.

We previously identified the *wrinkle free* mouse that carries a mutation resulting from insertion of a retrotransposon into *Slc27a4*, the gene encoding FATP4^17^. Mice lacking FATP4 (*Fatp4*^−/−^ mice) are born with thick, tight, “wrinkle free” skin and a dysfunctional skin barrier, and they die neonatally due to restricted movements and dehydration. *Fatp4*^+*/−*^ mice are phenotypically normal. Similar phenotypes have been reported in independent *Fatp4* mutants^18,19^. Expression of either a Fatp4 or a Fatp1 transgene in suprabasal keratinocytes restores the neonatal lethality and rescues the skin phenotype in *Fatp4* mutants, demonstrating common substrate preferences, enzymatic activities, and biological functions^14,20^.

Mutations disrupting human FATP4 are found in patients with ichthyosis prematurity syndrome, which manifests with premature birth, respiratory symptoms, and swollen skin with severe caseosa-like scaling^21–23^. Surviving patients recover and show a non-scaly ichthyosis during childhood with dry and pruritic skin and often with respiratory and/or food allergies^24^.

Epidermal lipid analyses of *Fatp4*^−/−^ newborn mice reveal a decreased level of ceramides carrying fatty acyl chains with 26 or more carbons and an increased level of those with 24 or fewer carbons^19,20^. Consistent with these results, *Fatp4* mutant epidermis displays an increased amount of FFA likely due to failure of mutant keratinocytes to activate longer chain FAs to an acyl-CoA form and accumulation of FFA inside cells^14^.

To investigate how the defect in FFA activation in *Fatp4*^−/−^ epidermis affects the incorporation of FAs into ceramides, we performed lipid analyses by thin layer chromatography (TLC) and electrospray ionization-mass spectrometry (ESI-MS) with free, extractable lipids and protein-bound lipids from newborn epidermis. We identified two lipid types previously speculated to be present in normal mice and identified abnormalities in several lipid types. *Fatp4*^−/−^ lipid abnormalities were remedied by suprabasal keratinocyte expression of either a Fatp4 or a Fatp1 transgene. Consistent with these results, ultrastructural analyses showed that transgene-derived FATP4 or FATP1 could ameliorate most of the defects seen in *Fatp4*^−/−^ epidermis, including the lamellar body secretory system and corneocyte lipid envelope formation. Our results provide insights into how FATP4 deficiency alter fatty acyl moieties in various barrier lipids and subsequently the permeability barrier function of the skin. Our data also support our previous speculation that FATP4 and FATP1 have overlapping roles in facilitating the usage of ultralong-chain FA (ULCFA; ≥ C25).

## Results

### Ultrastructural analyses of *Fatp4*^−/−^ skin

**Figure 1:**
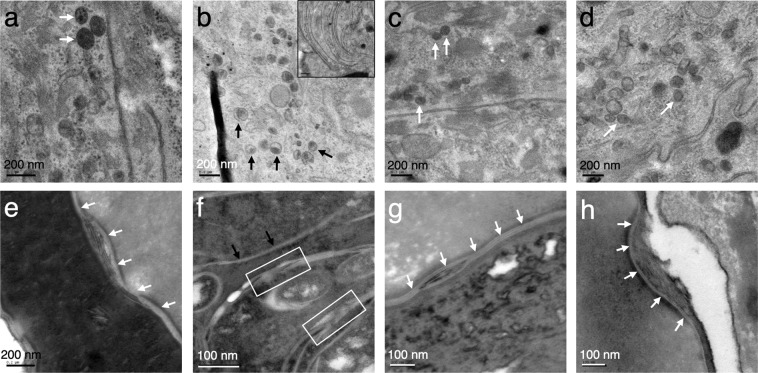
Defects in *Fatp4*^−/−^ lamellar body secretory system ultrastructure. Lamellar body structure ((**a**–**d**), reduced osmium tetroxide post-fixation) and extracellular lipid processing ((**e**–**h**), ruthenium tetroxide post-fixation) were evaluated by transmission electron microscopy. Typical abnormal curved membrane structures, seen only in *Fatp4*^−/−^ epidermis, are shown in the inset in (**b**). *Fatp4*^−/−^ lamellar bodies (**b**) were irregularly shaped and incompletely filled (black arrows) compared to control (**a**) and *Fatp4*^−/−^ rescued with FATP4 (**c**) or FATP1 (**d**) (white arrows show normal lamellar bodies). Secreted lipid in *Fatp4*^−/−^ was reduced in quantity, as seen by thin sheets of lamellar lipids ((**f**), black arrows), and exhibited extensive phase separation (white boxes) compared to control (**e**) and *Fatp4*^−/−^ rescued with FATP4 (**g**) or FATP1 (**h**) (white arrows show normally processed lamellar lipids). Representative images are shown from two mice of each genotype.

**Figure 2:**
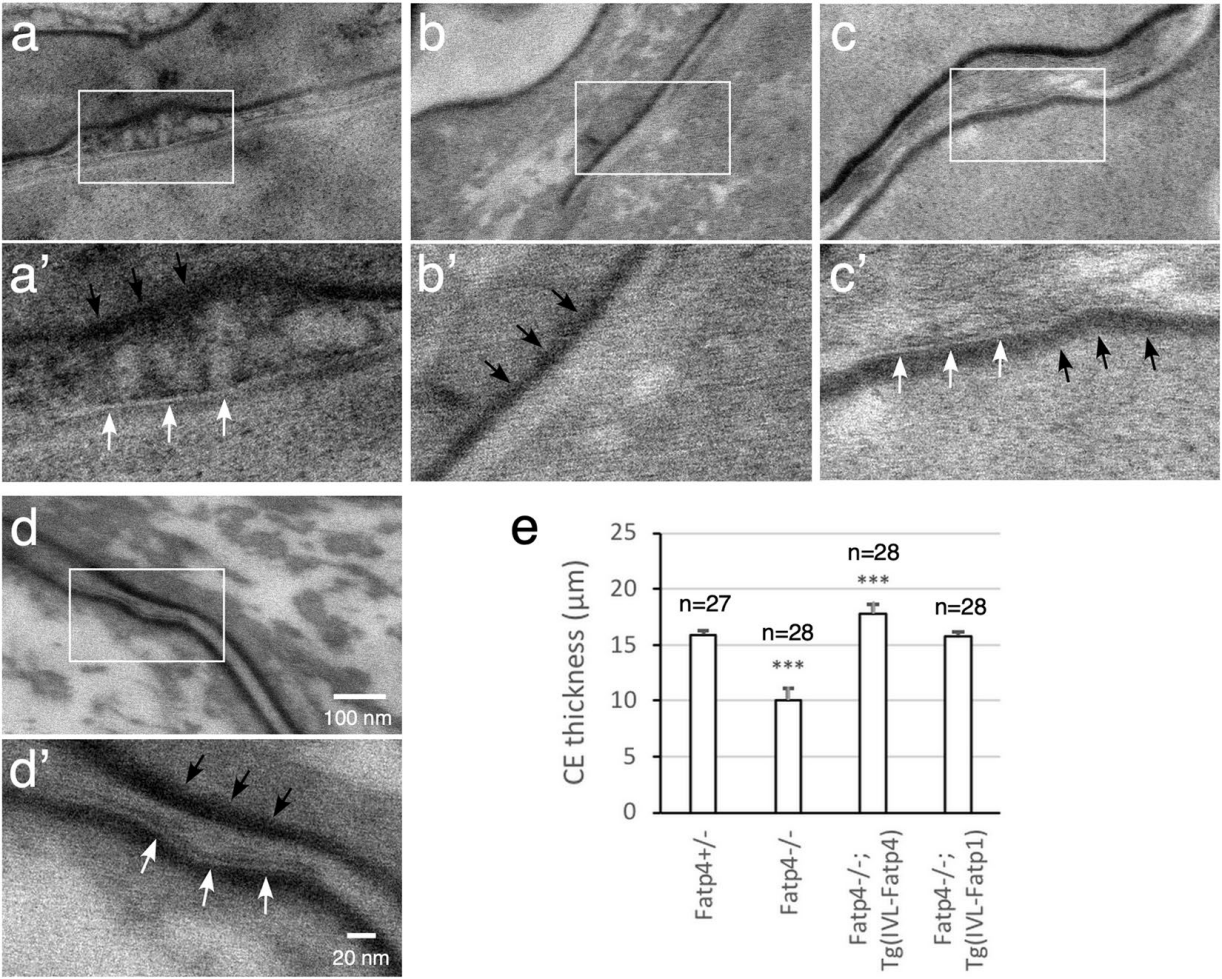
Defects in ultrastructure of Fatp4^−/−^ corneocyte lipid envelope (CLE) and cornified envelope (CE). The CLE and CE were examined by transmission electron microscopy as described in Materials and Methods. The boxed regions in **a** to **d** are magnified in **a’** to **d’**, respectively. White arrows indicate typical CLE morphology: a regular, 4 nm thick lucent band on the external face of the CE (black arrows). *Fatp4*^−/−^ stratum corneum (**b’**) was characterized by no visible CLE and a thin CE compared to control (**a’**), FATP4- (**c’**), and FATP1- (**d’**) rescued mice. Representative images are shown from two mice of each genotype. CE thickness was quantified (**e**) as described in Materials and Methods with the n numbers, standard deviation, and statistical significance indicated (***P < 0.001). IVL, involucrin promoter.

Ultrastructural analysis revealed curved multilamellar membrane-like structures in the granular and cornified layers of *Fatp4*^−/−^ newborn mice (Fig. 1b, inset) that are also present in ichthyosis prematurity syndrome patients^25^. *Fatp4*^−/−^ skin also showed multiple abnormalities in the lamellar body secretory system, including a higher density of lamellar bodies (Fig. 1b) with a decrease in the lamellar contents (black arrows) vs. controls (Fig. 1a, white arrows). In contrast, fusion of lamellar bodies to the plasma membrane, secretion of lipid contents to the extracellular space, and post-secretory processing appeared normal (data not shown). However, the normal-appearing lamellar bilayers were reduced in overall quantity and accompanied by extensive non-lamellar phase separation compared to controls (Fig. 1e,f). Transgenic FATP4 or FATP1 expression in mutant suprabasal keratinocytes rescued most of the ultrastructural abnormalities, with partially corrected lamellar body contents (Fig. 1c,d) and normalization of the quantity and structure of lamellar bilayers (Fig. 1g,h). *Fatp4* mutants showed virtually no visible corneocyte lipid envelope compared to controls (Fig. 2a’,b’), which was reverted by transgenic expression of FATP4 or FATP1 (Fig. 2c’,d’).

**Figure 3:**
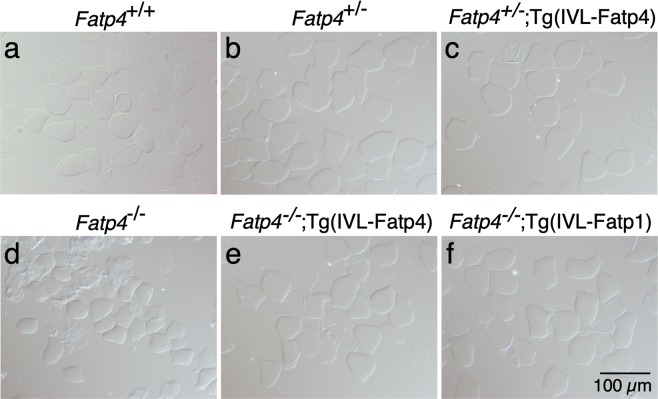
Defects in *Fatp4*^−/−^ cornified envelope (CE). The CE was isolated from the dorsal skin of 17.5-day embryos and viewed under a phase contrast microscope. The CE was smaller in *Fatp4* mutants (**d**) compared to controls (**a**–**c**) but was normalized by transgene-derived FATP4 (**e**) and FATP1 (**f**). Representative images are shown from at least two mice of each genotype. IVL, involucrin promoter.

Besides changes in lipid structures, *Fatp4* mutant skin showed a significant reduction in thickness of the protein cornified envelope compared to controls (quantification shown in Fig. 2e). Consistent with this, the cornified envelope remaining after boiling the skin with an ionic detergent and a reducing agent was smaller in *Fatp4* mutants from embryonic day 16.5 onwards (embryonic day 17.5 shown in Fig. 3d) compared to controls (Fig. 3a–c). Exogenous FATP4 and FATP1 ameliorated the cornified envelope size defect (Fig. 3e,f). Whereas FATP1 expression restored the cornified envelope thickness, FATP4 expression increased the cornified envelope thickness in *Fatp4* mutants to a degree that was slightly higher than controls (Fig. 2).

### Alterations of unbound lipids in *Fatp4*^−/−^ epidermis

**Figure 4:**
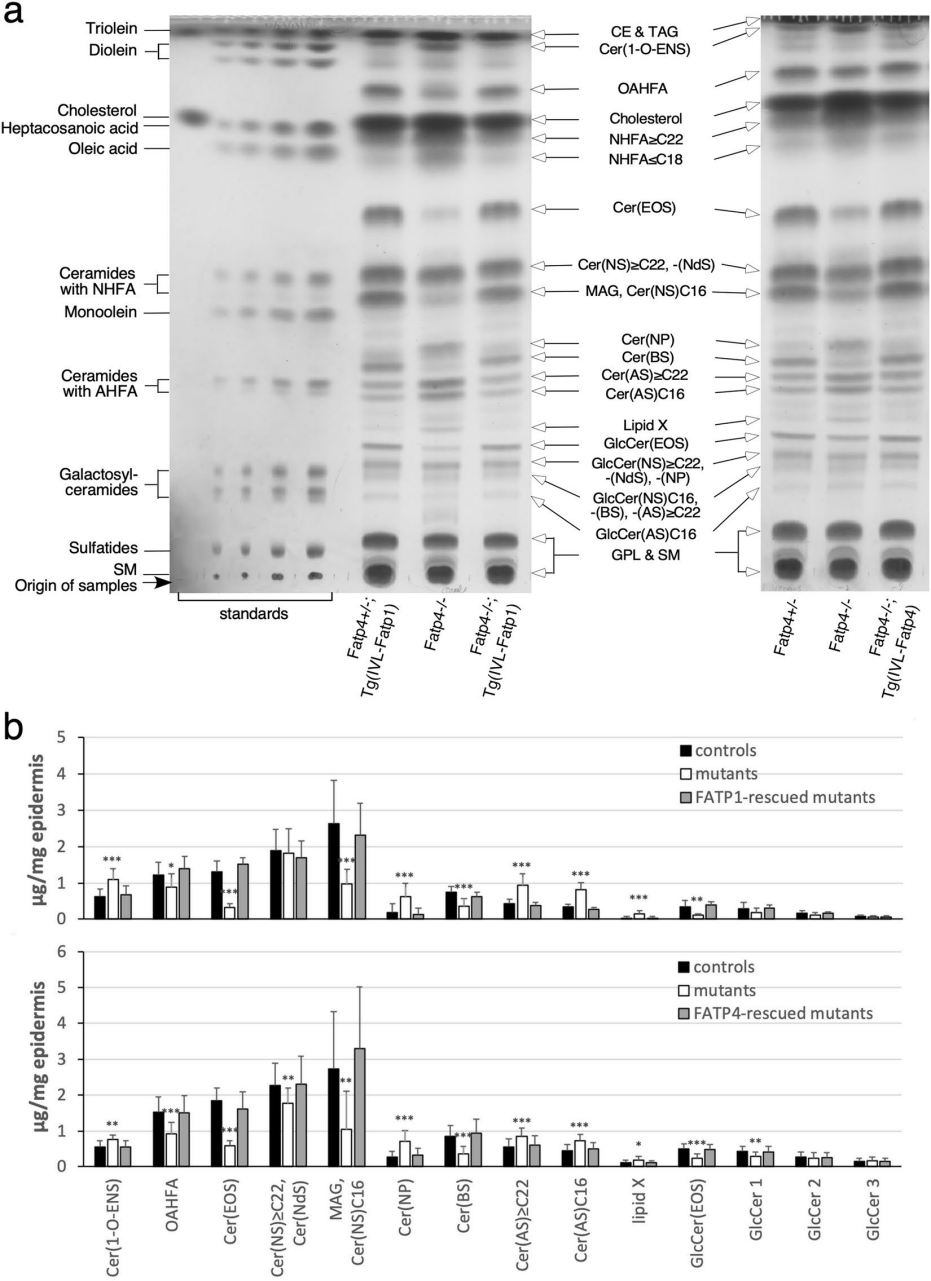
Alterations in the repertoire of unbound epidermal lipids in *Fatp4*^−/−^ mice. Free, extractable lipids from newborn mouse epidermis were co-resolved with standards by TLC (**a**) and quantified in µg per mg of dry epidermis (**b**). The decreased intensities of OAHFA, Cer(EOS), MAG/Cer(NS)C16, Cer(BS), and GlcCer(EOS) bands and the increased levels of Cer(1-O-ENS), Cer(NP), Cer(AS) ≥ C22, Cer(AS)C16, and lipid X observed in *Fatp4*^−/−^ mice were normalized by expression of Fatp1 (*Fatp4*^−/−^; Tg(IVL-Fatp1) in **a**, left; **b**, top) and Fatp4 (*Fatp4*^−/−^; Tg(IVL-Fatp4) in **a**, right; **b**, bottom) transgenes. Lipid standards (**a**, left) and unbound epidermal lipid types (arrows) are indicated. Quantification data (**b**) were obtained from two separate studies with standard deviation and statistical significance indicated (*P < 0.05; **P < 0.01; ***P < 0.001). Each study contained 4 each of control, *Fatp4*^−/−^, and transgene-rescued mice. Glycerophospholipids (GPL), sphingomyelin (SM), and the previously reported cholesteryl ester (CE), triacylglycerol (TAG), FAs, and cholesterol^14^ are not included in **b**. GlcCer 1, 2, and 3 refer to the three bands running immediately behind the GlcCer(EOS) band shown in (**a**). The TLC results shown in a are from two separate TLC experiments; full vertical lengths are shown. IVL, involucrin promoter.

To resolve how the defect in FA activation in *Fatp4*^−/−^ epidermis affected the incorporation of FAs into ceramides, we performed lipid analyses by TLC and high-resolution ESI-MS with both free, extractable lipids and protein-bound lipids from newborn epidermis. Consistent with previous reports, our TLC analyses of controls showed five major ceramide bands in the free, extractable lipid fraction of the epidermis (Fig. 4a). After recovery from the TLC plate for MS, the five ceramide bands were verified to be: 1) ω-O-acylceramide (Cer(EOS)); 2) a mixture of sphingosine non-hydroxyceramide with a fatty acyl chain of at least 22 carbons (Cer(NS) ≥ C22) and dihydrosphingosine non-hydroxyceramide (Cer(NdS)); 3) sphingosine β-hydroxyceramide (Cer(BS)); 4) sphingosine α-hydroxyceramide with a fatty acyl chain of at least 22 carbons (Cer(AS) ≥ C22); and 5) Cer(AS) with an acyl chain of 16 carbons (Cer(AS)C16). The Cer(BS) band was previously indicated as phytosphingosine (4-hydroxysphinganine) non-hydroxyceramide (Cer(NP))^26^, but with ESI-MS we verified this band to be Cer(BS) as found in human stratum corneum^27^, in agreement with original speculation^28^.

Compared to controls, *Fatp4*^−/−^ epidermis displayed abnormal patterns of several ceramide classes including significant reduction in Cer(EOS) and Cer(BS) (Fig. 4a,b). Cer(EOS) contains an amide-linked ULCFA with a terminal ω-hydroxy group that is further esterified with an additional FA, mainly linoleic acid, and is thought to be critical for barrier function^29,30^. Quantification of Cer(EOS) in two separate studies revealed significant decreases in *Fatp4*^−/−^ epidermis. The reduction in Cer(EOS) was accompanied by a significantly reduced level of a special FA that migrated immediately ahead of the cholesterol band on TLC with a solvent system for polar lipids (Fig. 4a). This FA band was identified previously to be O-acyl-ω-hydroxy FA (OAHFA)^31^, a unique type of ULCFA that constitutes the fatty acyl moiety in Cer(EOS). Its identity was verified here by ESI-MS.

*Fatp4*^−/−^ epidermis showed a discrete band migrating immediately ahead of Cer(BS) that was barely visible in controls (Fig. 4a). ESI-MS identified this to be Cer(NP). *Fatp4*^−/−^ epidermis also showed significantly increased levels of both Cer(AS) ≥ C22 and Cer(AS)C16 (Fig. 4a,b). In contrast, the amount of the band containing Cer(NS) ≥ C22 and Cer(NdS) was reduced significantly in *Fatp4*^−/−^ epidermis in one study but unchanged in the other study (Fig. 4b).

Whereas ceramides in control epidermis were resolved into five major bands on TLC, their glycosylated precursors, glucosylceramides (GlcCer), stored in lamellar bodies of granulocytes were separated into four bands (Fig. 4a). With ESI-MS we verified the least and the most polar bands of the four to be GlcCer(EOS) and GlcCer(AS)C16, respectively, as shown previously^26,28,32^. The other two GlcCer bands were identified to be a mixture of GlcCer(NS) ≥ C22, GlcCer(NdS), and GlcCer(NP), and a mixture of GlcCer(NS)C16, GlcCer(BS), and GlcCer(AS) ≥ C22. In agreement with the reduced Cer(EOS), *Fatp4*^−/−^ epidermis also showed a reduced amount of GlcCer(EOS) (Fig. 4a,b). Quantification of other GlcCer bands did not reveal significant changes in mutants except reduced GlcCer(NS) ≥ C22, GlcCer(NdS), and GlcCer(NP) mixture (GlcCer 1 in Fig. 4b) in *Fatp4*^−/−^ epidermis in the same study that showed decreases in Cer(NS) ≥ C22 and Cer(NdS).

In addition to these ceramide and GlcCer classes, mouse epidermal lipids normally contains a less polar ceramide with an ester-linked FA attached to the 1-hydroxyl group of the long-chain base (Fig. 4a)^28^. By ESI-MS analysis of bands extracted from the TLC plate we recently identified this ceramide ester to be 1-O-acylceramide (Cer(1-O-ENS)^33^, consistent with a previous report^34^. *Fatp4*^−/−^ epidermis showed increased levels of Cer(1-O-ENS) and an unknown ceramide band (Lipid X) that migrated immediately ahead of the GlcCer(EOS) band (Fig. 4a,b). *Fatp4*^−/−^ epidermis also showed a dramatically reduced amount of a band that migrated immediately behind the Cer(NS) band (Fig. 4a,b). By MS we identified this to be mainly monoacylglycerol (MAG)^35^, in agreement with original speculation^32^. This band contained Cer(NS)C16 as a minor component, in contrast to a previous report that it was a major component^26^. In *Fatp4*^−/−^ mice expressing either transgenic FATP1 or FATP4 (Fig. 4b), the abnormalities observed in unbound lipids were all remedied.

### Alterations of protein-bound lipids in *Fatp4*^−/−^ epidermis

**Figure 5:**
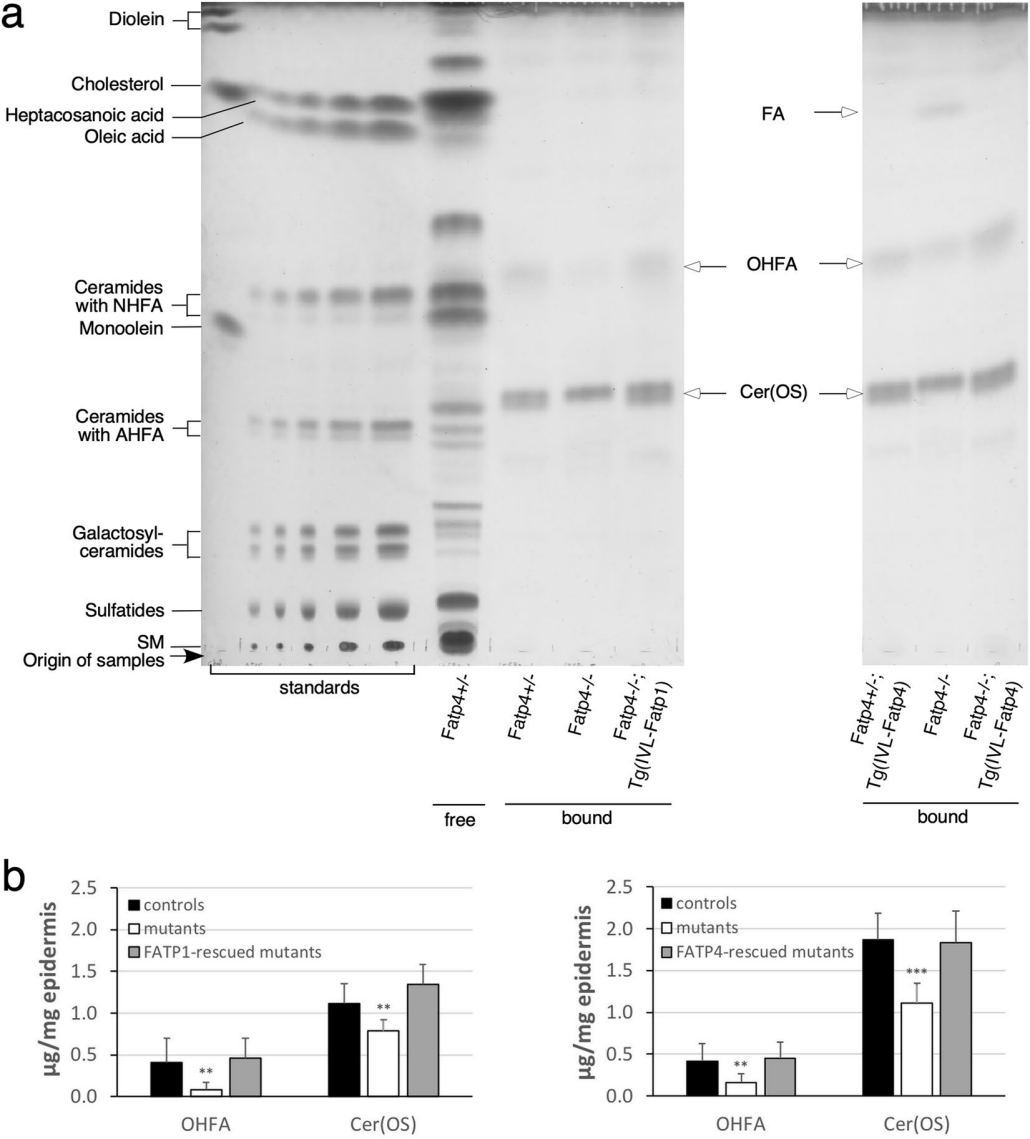
Alterations in the repertoire of protein-bound epidermal lipids in *Fatp4*^−/−^ mice. Protein-bound lipids from newborn mouse epidermis were co-resolved by TLC with standards and free epidermal lipids (**a**) and quantified in µg per mg of dry epidermis (**b**). The decreased levels of OHFA and Cer(OS) seen in *Fatp4*^−/−^ mice were normalized by the Fatp1 (*Fatp4*^−/−^; Tg(IVL-Fatp1) in **a**, left; **b**, left) and Fatp4 (*Fatp4*^−/−^; Tg(IVL-Fatp4) in **a**, right; **b**, right) transgenes. A faster migrating FA band was seen in some *Fatp4*^−/−^ samples. Lipid standards (**a**, left) and protein-bound epidermal lipids (arrows) are indicated. Quantification data (**b**) were obtained from two separate studies with standard deviation and statistical significance indicated (**P < 0.01; ***P < 0.001). Each study contained 6 each of control, *Fatp4*^−/−^, and transgene-rescued mutant mice. The TLC results shown in **a** are from two separate TLC experiments; full vertical lengths are shown. IVL, involucrin promoter.

TLC analysis revealed two major protein-bound lipid bands in epidermal extracts from control newborns (Fig. 5a). By MS they were identified as sphingosine ω-hydroxyceramide (Cer(OS)) and ω-hydroxy FAs (OHFA), the ULCFA that constitutes the fatty acyl moiety in protein-bound Cer(OS), in agreement with other reports^26,36^. Mutants displayed reduced levels of both protein-bound Cer(OS) and OHFA (Fig. 5b). Another faster migrating FA band reported by others was detected in some of the *Fatp4*^−/−^ samples examined but not in controls (right panel of Fig. 5a). The abnormalities in Cer(OS) and OHFA were remedied by epidermal FATP1 or FATP4 expression (Fig. 5b).

### Alterations of FFA and fatty acyl moieties in unbound lipids of *Fatp4*^−/−^ epidermis

#### Free fatty acid

**Figure 6:**
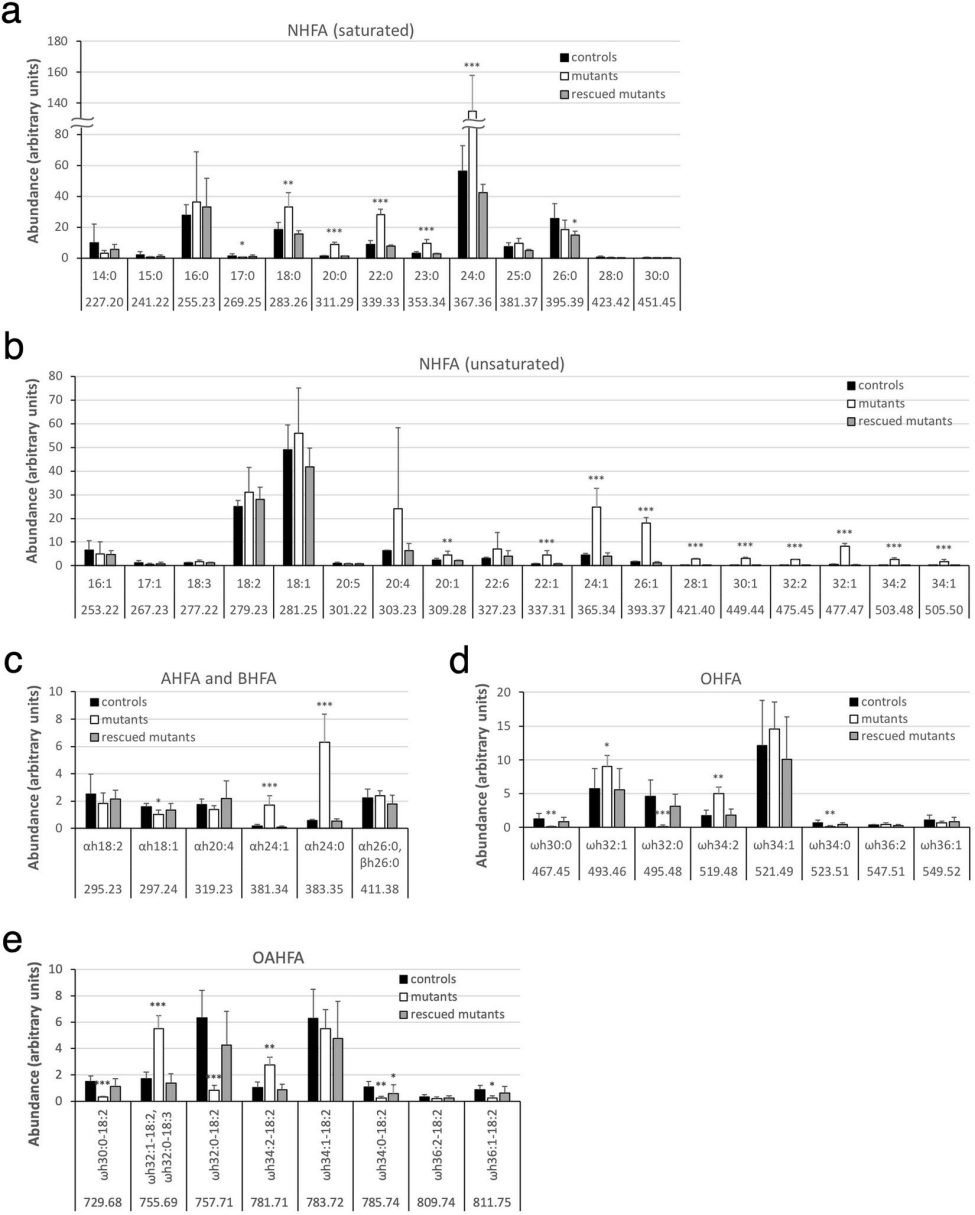
Alterations in FFA content in unbound epidermal lipids in *Fatp4*^−/−^ mice. Pooled fractions 4 and 5 of unbound epidermal lipids of newborns were analyzed by ESI-MS as [M − H]^−^ ions in the negative-ion mode and quantified in arbitrary units. *Fatp4*^−/−^ mice showed increased levels of saturated NHFA (C18-C24) and monounsaturated NHFA (C20-C34) (**a**,**b**), decreased levels of saturated OHFA and the OAHFA with a saturated fatty acyl backbone (**d**,**e**), and abnormal levels of AHFA and BHFA (**c**). Almost all these defects were normalized by the Fatp1 transgene. Data were obtained from 4 each of *Fatp4*^+*/−*^ (controls), *Fatp4*^−/−^, and FATP1-rescued mutants with m/z ratios, standard deviation, and statistical significance shown (*P < 0.05; **P < 0.01; ***P < 0.001). See Materials and Methods for nomenclature.

We previously showed that *Fatp4*^−/−^ epidermis displayed an increased amount of FFA in the unbound lipid fraction likely resulting from *Fatp4*^−/−^ keratinocytes’ failure to activate ULCFA to an acyl-CoA form, with subsequent accumulation of FFA inside cells^14^. To investigate the spectrum of the elevated FFA pool and how the incorporation of FAs into unbound lipids was affected in *Fatp4*^−/−^ epidermis, unbound lipid extract was separated with solid-phase extraction columns into FFA and various lipid fractions and subjected to high-resolution ESI-MS (Figs S1, S2, S3). The results showed that the elevated FFA pool (fractions 4 and 5 in Materials and Methods) in *Fatp4*^−/−^ epidermis was mainly composed of saturated, non-hydroxy FAs (NHFA) containing 18 to 24 carbons (Fig. 6a) and monounsaturated NHFA containing 20 to 34 carbons (Fig. 6b). The amounts of both saturated and unsaturated α-hydroxy FAs (AHFA) containing 24 carbons were also dramatically increased (Fig. 6c). In contrast, the proportions of saturated OHFA (Fig. 6d) and the OAHFA that carried a saturated fatty acyl backbone (Fig. 6e) were dramatically decreased; examples of OHFA and OAHFA identification are shown in Figs S4, S5.

#### Non-hydroxyceramides

**Figure 7:**
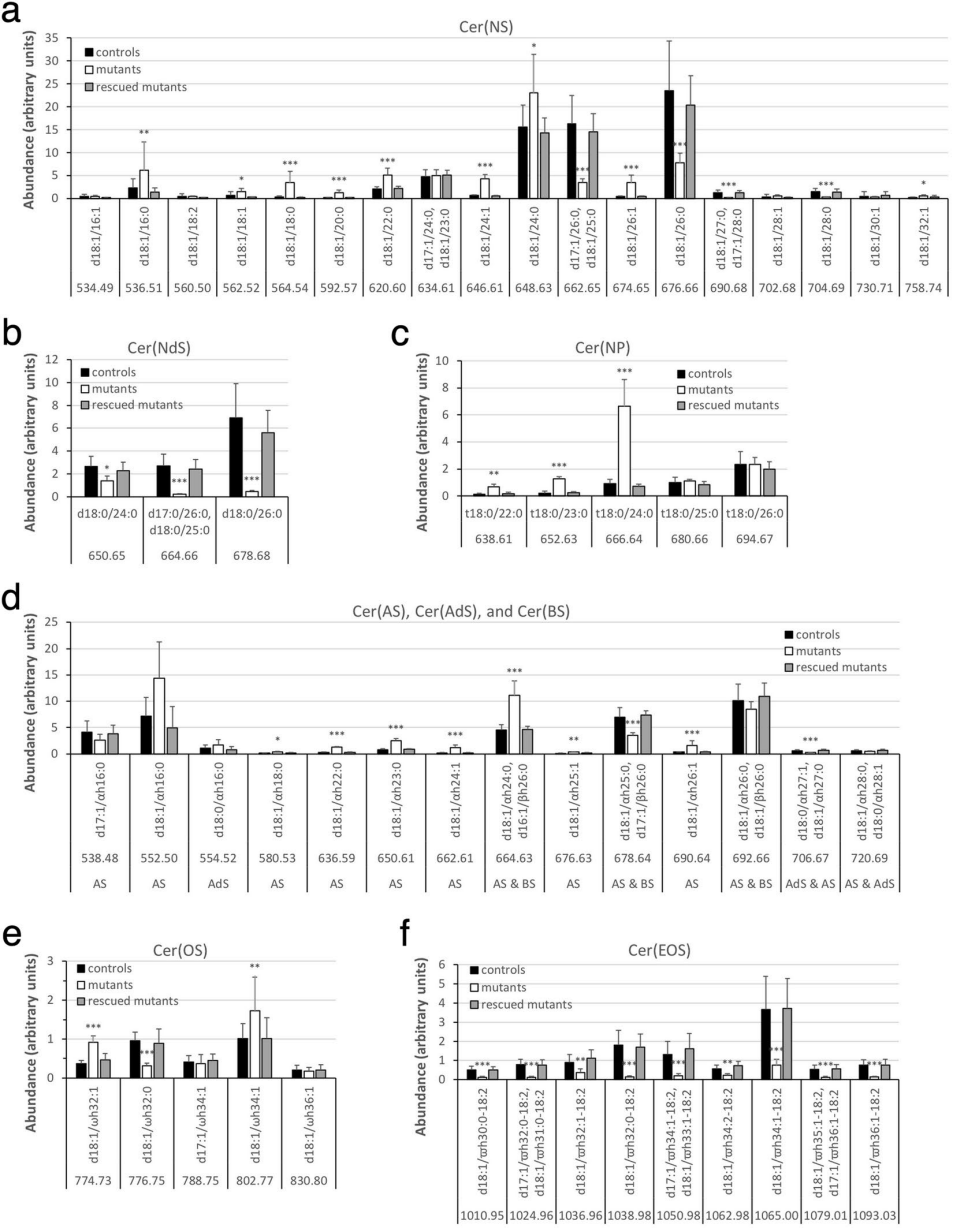
Alterations in ceramide content in unbound epidermal lipids in *Fatp4*^−/−^ mice. Fraction 3 of unbound epidermal lipids of newborn mice was analyzed by ESI-MS as [M − H]^−^ ions in the negative-ion mode and quantified in arbitrary units. *Fatp4*^−/−^ mice showed a shift to the Cer(NS) species with a shorter saturated acyl chain (**a**), decreased levels of the Cer(NdS) and Cer(OS) species that contained a saturated acyl chain (**b**,**e**) and of all Cer(EOS) species (**f**), and increased levels of several species in Cer(NP) and Cer(AS) (**c**,**d**). These defects were all normalized by the Fatp1 transgene. Data were obtained from 4 each of *Fatp4*^+*/−*^ (controls), *Fatp4*^−/−^, and FATP1-rescued mutants with m/z ratios, standard deviation, and statistical significance shown (*P < 0.05; **P < 0.01; ***P < 0.001). See Materials and Methods for nomenclature.

MS of fraction 3 of unbound lipids from control epidermis identified Cer(NS) class carrying an acyl chain of 16 to 32 carbons (Fig. 7a; Fig. S6) and Cer(NdS) class carrying an acyl chain of 24 to 26 carbons (Fig. 7b; Fig. S7). Whereas by TLC the band containing these two ceramide classes in *Fatp4*^−/−^ epidermis showed an altered level in only one of our two studies, Cer(NS) displayed altered acyl chain length distribution by MS. *Fatp4*^−/−^ epidermis showed a significantly decreased proportion of Cer(NS) with a saturated acyl moiety containing 25 or more carbons; e.g., two major Cer(NS) species, d17:1/26:0 and d18:1/26:0, were reduced with 4.7- and 3.0-fold changes, respectively. In contrast, *Fatp4*^−/−^ epidermis showed increased proportions of Cer(NS) species containing either a shorter saturated acyl chain or an unsaturated acyl chain. All Cer(NdS) species identified in control epidermis carried a saturated acyl chain, and their levels were decreased in *Fatp4*^−/−^ epidermis, especially d18:0/26:0, which showed a 16.0-fold change. The minor lipid Cer(NP) identified in controls comprised a saturated acyl chain carrying 22 to 26 carbons. The increased Cer(NP) in mutants was reflected in species carrying an acyl moiety of 22 to 24 carbons (Fig. 7c; Fig. S8).

#### Hydroxyceramides

By TLC *Fatp4*^−/−^ epidermis showed decreased Cer(BS) and increased levels of two relatively polar ceramide classes, Cer(AS) ≥ C22 and Cer(AS)C16, in the unbound lipid fraction. MS identified three Cer(BS) species in fraction 3 from controls, all carrying the same ß-hydroxy FA (BHFA) 26:0 but each with a different long-chain base d16:1, d17:1, or d18:1 (Fig. 7d). Cer(AS) in controls contained an AHFA chain carrying 16 to 28 carbons (Fig. 7d). All three Cer(BS) species identified shared the same m/z ratios with their corresponding Cer(AS) isomers, e.g., d18:1/βh26:0 & d18:1/αh26:0 both at an m/z ratio of 692.66 when detected as [M - H]^−^ ions, making it difficult to assess the exact changes in these ceramides individually in mutants. Of the other Cer(AS) species that did not have m/z ratios the same as Cer(BS), several were increased in mutants, such as d18:1/αh22:0 and d18:1/αh26:1. By TLC *Fatp4*^−/−^ epidermis displayed increased Cer(AS)C16; however, by MS the changes in amounts of the two individual Cer(AS)C16 species identified were not statistically significant. MS of fraction 3 also revealed Cer(AdS) and Cer(OS) as minor ceramide classes in control (Fig. 7d,e; Fig. S9). The only Cer(OS) species that carried a saturated acyl chain, d18:1/ωh32:0, was decreased in abundance in *Fatp4*^−/−^ epidermis.

#### ω-O-acylceramide

From fraction 3 of unbound lipids from control, MS also identified the Cer(EOS) class carrying 30 to 36 carbons in the OHFA backbone (Fig. 7f). As reported by others, the OHFA moiety of Cer(EOS) was esterified mainly with linoleic acid (18:2). The reduced Cer(EOS) in *Fatp4*^−/−^ epidermis detected by TLC was attributed to reduced Cer(EOS) species carrying a saturated OHFA backbone and those carrying an unsaturated OHFA backbone. For example, the two most abundant species in control mice, d18:1/ϖh34:1-18:2 and d18:1/ϖh32:0-18:2, showed decreased proportions in *Fatp4*^−/−^ epidermis with 4.8- and 11.9-fold changes, respectively.

#### Monoacylglyerol

**Figure 8:**
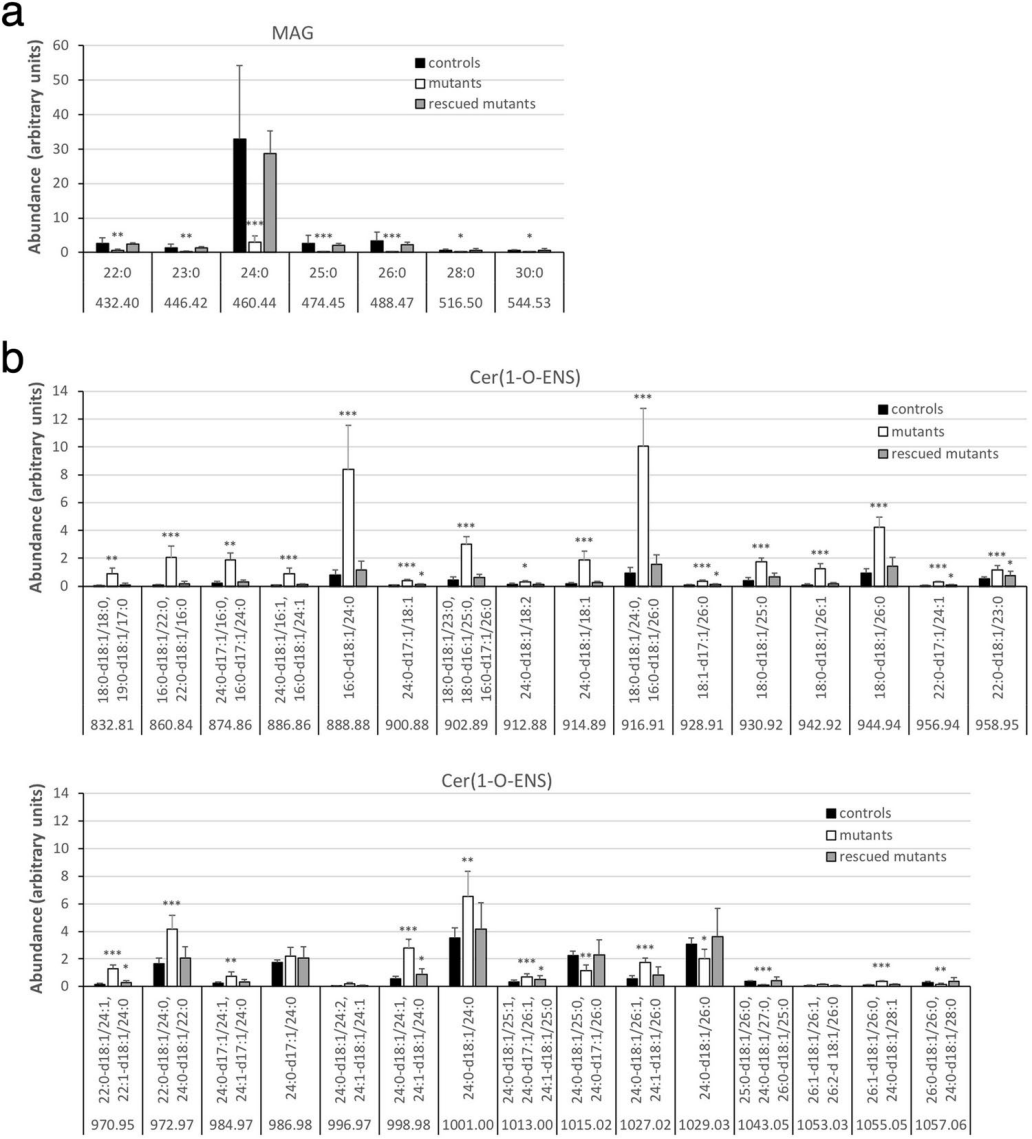
Alterations in MAG and 1-O-acylceramide content in unbound epidermal lipids in *Fatp4*^−/−^ mice. Fractions 3 and 2 of unbound epidermal lipids of newborn mice were analyzed by ESI-MS in the positive-ion mode as [M + NH_4_]^+^ ions (**a**) and as [M + H]^+^ ions (**b**), respectively, and quantified in arbitrary units. The decreased levels of MAG species seen in *Fatp4*^−/−^ mice were all normalized by the Fatp1 transgene (**a**). *Fatp4*^−/−^ mice showed increased levels of many Cer(1-O-ENS) species that carried 48 or fewer total carbons in their two acyl chains, almost all of which were normalized by the Fatp1 transgene (**b**). Data were obtained from 4 each of *Fatp4*^+*/−*^ (controls), *Fatp4*^−/−^, and FATP1-rescued mutants with m/z ratios, standard deviation, and statistical significance shown (*P < 0.05; **P < 0.01; ***P < 0.001). See Materials and Methods for nomenclature. MAG species are listed by their acyl groups. Cer(1-O-ENS) species at the same m/z ratio are listed in descending order in amount found in controls.

In addition to ceramide classes, MS identified several MAG species in fraction 3 of unbound lipids from control epidermis. They carried a saturated fatty acyl chain ranging from 22 to 30 carbons with 24:0 being the dominant one (Fig. 8a), consistent with a previous report^32^. *Fatp4*^−/−^ epidermis showed dramatic reductions in levels of all MAG species.

#### 1-O-acylceramide

Cer(1-O-ENS), the least polar ceramide in our study, was identified by MS in fraction 2 from control epidermis (Fig. 8b). The increase in Cer(1-O-ENS) level in *Fatp4*^−/−^ epidermis by TLC was reflected by the drastically increased levels of many of those carrying 48 or fewer total carbons in their two acyl chains. In contrast, *Fatp4*^−/−^ epidermis displayed significantly decreased levels of those with two saturated, longer acyl chains, e.g., 25:0-d18:1/26:0.

#### Glucosylceramide

**Figure 9:**
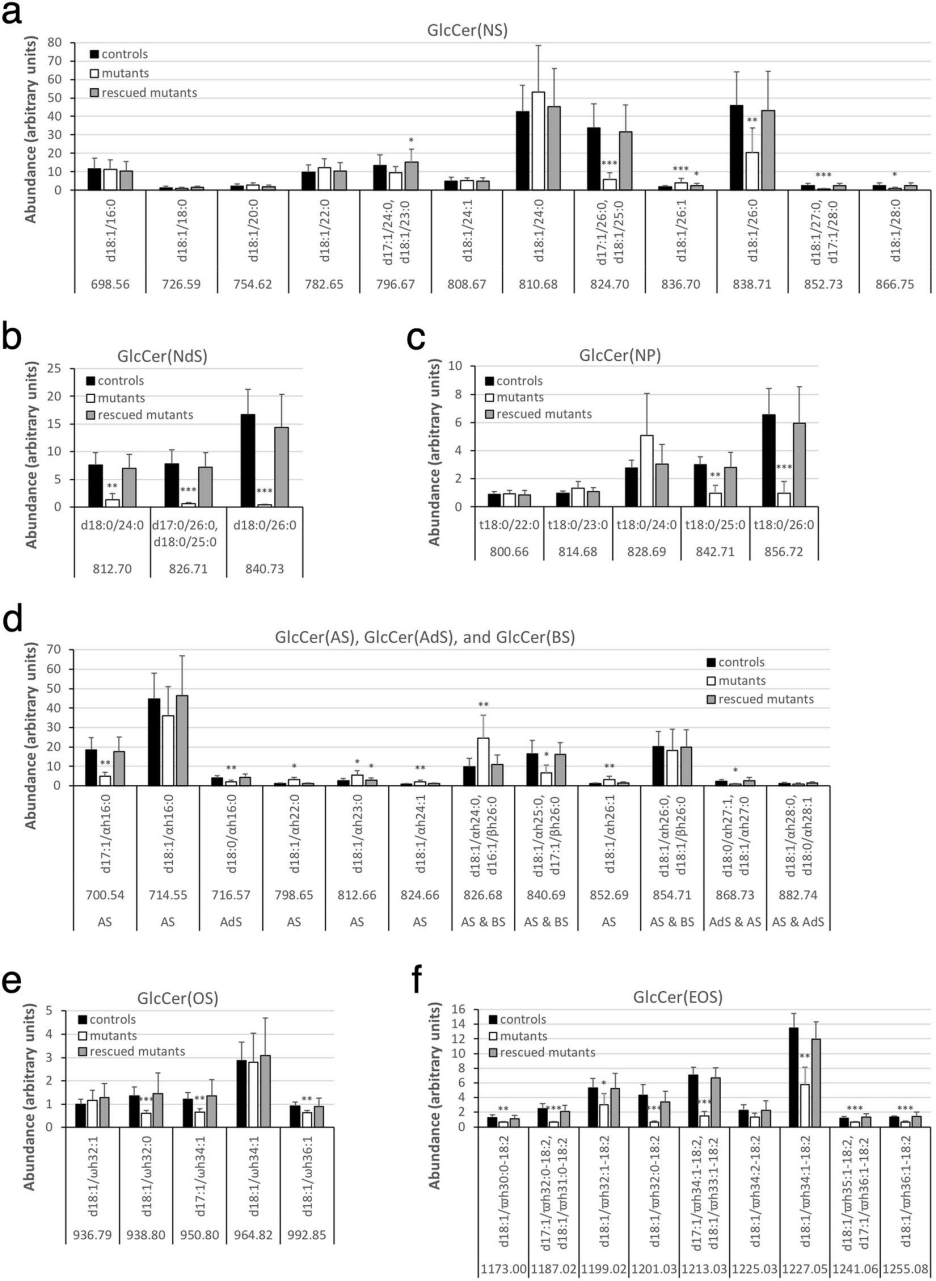
Alterations in GlcCer content in unbound epidermal lipids in *Fatp4*^−/−^ mice. Fraction 6 of unbound epidermal lipids of newborn mice was analyzed by ESI-MS as [M − H]^−^ ions in the negative-ion mode and quantified in arbitrary units. *Fatp4*^−/−^ mice showed decreased levels of the GlcCer(NS), GlcCer(NdS), and GlcCer(NP) species that contained a longer saturated acyl chain (**a**,**b**,**c**), of several GlcCer(OS) species (**e**), and of nearly all GlcCer(EOS) species (**f**), and increased levels of several GlcCer(AS) species (**d**). Almost all these defects were normalized by the Fatp1 transgene. Data were obtained from 5 each of *Fatp4*^+*/−*^ (controls), *Fatp4*^−/−^, and FATP1-rescued mutants with m/z ratios, standard deviation, and statistical significance shown (*P < 0.05; **P < 0.01; ***P < 0.001). See Materials and Methods for nomenclature.

 MS on fraction 6 of unbound lipids from control showed glycosylated precursors of most ceramide species identified in our study (Fig. S10). All three GlcCer(NdS) species (Fig. 9b) and nearly all GlcCer(EOS) species (Fig. 9f) were reduced in *Fatp4*^−/−^ epidermis, comparable to the reductions seen in the corresponding Cer(EOS) species. For example, as the GlcCer(EOS) species d18:1/ϖh32:0-18:2 showed the most dramatic reduction (6.6-fold changes) in mutants, its corresponding processed Cer(EOS) species also showed the most dramatic reduction (11.9-fold changes). The proportions of the several GlcCer(AS) species with a distinct m/z ratio showed similar changes to their corresponding Cer(AS) species except those carrying an acyl chain of 16 carbons (Fig. 9d). In contrast, the changes in abundance of other GlcCer classes did not show good correlation with those of the corresponding processed forms of Cer. For example, whereas data on GlcCer(NS) in mutants reflected the significantly decreased proportions of Cer(NS) species with a saturated fatty acyl moiety containing 25 or more carbons, they did not reveal the corresponding increased proportions of Cer(NS) species containing an unsaturated acyl chain or a shorter saturated acyl chain (Fig. 9a). Also *Fatp4*^−/−^ epidermis showed decreased GlcCer(NP) species carrying a saturated acyl moiety with 25 or more carbons, in contrast to unchanged abundance of their corresponding Cer(NP) species (Fig. 9c).

Transgenic FATP1 expression in granulocytes ameliorated nearly all the compositional abnormalities detected in unbound epidermal lipids of *Fatp4*^−/−^ mice (Figs 6–9).

### Alterations of OHFA and fatty acyl moieties in protein-bound lipids in *Fatp4*^−/−^ epidermis

**Figure 10:**
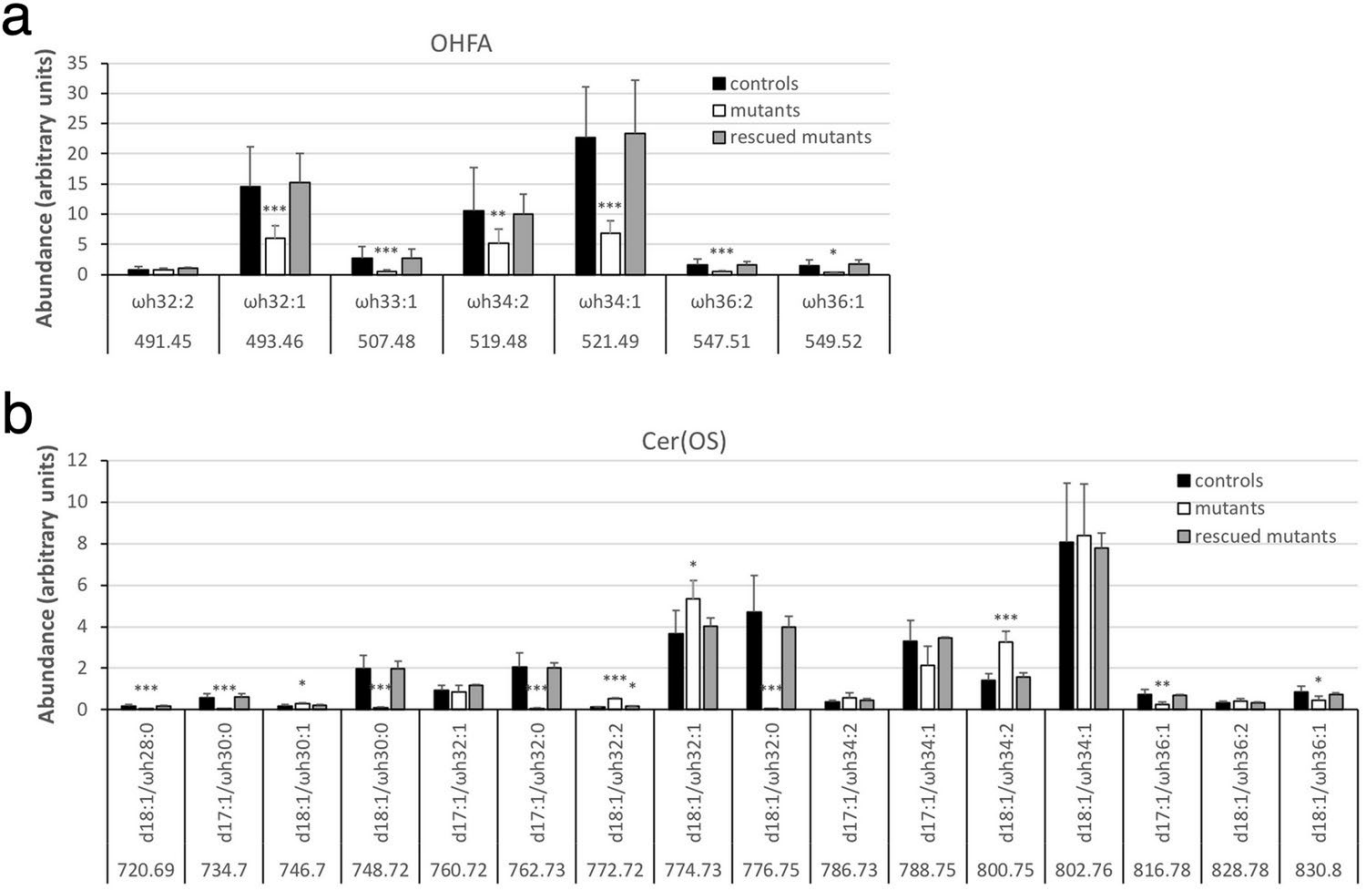
Alterations in FA and ceramide content in protein-bound epidermal lipids in *Fatp4*^−/−^ mice. Pooled fractions 4 and 5 (**a**) and fraction 3 (**b**) of protein-bound epidermal lipids of newborn mice were analyzed by ESI-MS as [M − H]^−^ ions in the negative-ion mode and quantified in arbitrary units. The decreased levels of nearly all OHFA species (**a**) and all saturated Cer(OS) species (**b**) seen in *Fatp4*^−/−^ mice were mostly normalized by the Fatp1 transgene. Data were obtained from 4 each of *Fatp4*^+*/−*^ (controls), *Fatp4*^−/−^, and FATP1-rescued mutants with m/z ratios, standard deviation, and statistical significance shown (*P < 0.05; **P < 0.01; ***P < 0.001). See Materials and Methods for nomenclature.

MS of pooled fractions 4 and 5 of protein-bound lipids from control epidermis identified OHFA containing 32 to 36 carbons, all carrying one or two unsaturated bonds with ωh34:1 and ωh32:1 being the most abundant (Fig. 10a). *Fatp4*^−/−^ epidermis displayed significantly decreased proportions of nearly all OHFA species. MS of fraction 3 of protein-bound lipids from control epidermis revealed Cer(OS) species carrying a saturated or unsaturated OHFA chain ranging from 28 to 36 carbons (Fig. 10b), in contrast to the protein-bound OHFA that all carried one or two unsaturated bonds. *Fatp4*^−/−^ epidermis displayed diminished levels of all saturated Cer(OS) species, especially d18:1/ωh32:0, which showed an over 170-fold decrease in abundance, and increased or unchanged levels of unsaturated species. Transgenic FATP1 expression in granulocytes normalized nearly all the compositional irregularities observed in protein-bound epidermal lipids of *Fatp4*^−/−^ mice (Fig. 10).

## Discussion

*Fatp4*^−/−^ epidermis displayed multiple ultrastructural anomalies in the lamellar body secretory system, a defective corneocyte lipid envelope, and a thinned cornified envelope. These anomalies reflect the lipid composition changes observed in both the unbound and protein-bound epidermal lipids of *Fatp4*^−/−^ mice, which normally constitute the intercellular lipid lamellae and the corneocyte lipid envelope, respectively.

Our TLC and ESI-MS analyses of the unbound fraction of epidermal lipids from normal newborn mice identified five major ceramide classes, Cer(EOS), Cer(NS), Cer(NdS), Cer(BS), Cer(AS), and three minor classes, Cer(1-O-ENS), Cer(NP), and Cer(OS). Our study is unique in that we identified Cer(BS) and the non-ceramide lipid MAG, compared to what has been reported previously^26,28,32,34^. Epidermal Cer(BS) and MAG were first reported in newborn mice nearly three decades ago based on TLC and chemical reactivities^28^ but were not verified by MS until now. Our quantification results for unbound lipids showed that *Fatp4*^−/−^ newborn epidermis displayed dramatically decreased proportions of OAHFA, Cer(EOS), MAG/Cer(NS)C16, Cer(BS), and GlcCer(EOS), and increased proportions of Cer(1-O-ENS), Cer(NP), Cer(AS) ≥ C22, Cer(AS)C16, and the unknown lipid X. On the other hand, protein-bound lipids from *Fatp4*^−/−^ epidermis displayed dramatically decreased proportions in both OHFA and Cer(OS), two major components of the corneocyte lipid envelope.

Our analysis of the unbound lipid fraction also revealed alterations in ceramide composition in *Fatp4*^−/−^ epidermis (See Summary in Table 1). *Fatp4*^−/−^ epidermis showed profound reductions in production of all Cer(EOS) species that carried a saturated or unsaturated OHFA backbone of at least 30 carbons. Second, changes in several other ceramide classes displayed a switch to a composition with decreased abundance of ceramides containing a saturated acyl chain of at least 25 carbons and with increased abundance of ceramides containing a shorter, saturated acyl chain. Taken together, our results suggest a less hydrophobic composition of the permeability barrier structure in *Fatp4*^−/−^ epidermis. Our data also demonstrate crucial roles for FATP4 in the uptake and/or activation of saturated NHFA and BHFA with at least 25 carbons and of saturated or unsaturated OHFA with at least 30 carbons for subsequent synthesis of their corresponding classes of ceramides. For example, FATP4 activates OHFA into a CoA form following the cytochrome P450 CYP4F22-catalyzed ω-hydroxylation of FAs that have been lengthened by elongation of VLCFA protein 4 (ELOVL4), as hypothesized for the production of Cer(EOS)^25,37,38^.

**Table 1:** Summarized alterations in fatty acyl chain length and saturation in unbound and protein-bound epidermal lipids in *Fatp4*^−/−^ mice.

	Fraction number	Lipid class	Acyl chain length^a^	Changes in Fatp4^−/−^			
				Saturated		Unsaturated	
				≥C25	<C25	≥C25	<C25
Unbound FAs	4 & 5	NHFA	C14-C34	≅	↑^e^	↑	↑^e^
		AHFA	C18-C26	?	↑	n.a.	varied
		BHFA	C26	?	n.a.	n.a.	n.a.
		OHFA	C30-C36	↓	n.a.	↑^e^	n.a.
		OAHFA	C30-C36	↓	n.a.	↑^e^	n.a.
Unbound lipids	3	Cer(NS)	C16-C32	↓	↑	↑^e^	↑^e^
		Cer(NdS)	C24-C26	↓	↓	n.a.	n.a.
		Cer(NP)	C22-C26	≅	↑	n.a.	n.a.
		Cer(AS)	C16-C28	?	↑^d^	↑	↑
		Cer(AdS)	C16, C27, C28	n.a.	≅	?	n.a.
		Cer(BS)	C26	↓^c^	n.a.	n.a.	n.a.
		Cer(OS)	C32-C36	↓	n.a.	↑^e^	n.a.
		Cer(EOS)	C30-C36	↓	n.a.	↓	n.a.
	6	GlcCer(NS)	C16-C28	↓	≅	↑	≅
		GlcCer(NdS)	C24-C26	↓	↓	n.a.	n.a.
		GlcCer(NP)	C22-C26	↓	≅	n.a.	n.a.
		GlcCer(AS)	C16-C28	?	↑^d^	↑	↑
		GlcCer(AdS)	C16, C27, C28	n.a.	↓	?	n.a.
		GlcCer(BS)	C26	?	n.a.	n.a.	n.a.
		GlcCer(OS)	C32-C36	↓	n.a.	↓^e^	n.a.
		GlcCer(EOS)	C30-C36	↓	n.a.	↓	n.a.
	2	1-O-Cer(ENS)	C16-C28; C16-C26^b^	↓	↑^e^	↑^e^	↑
	3	MAG	C22-C30	↓	↓	n.a.	n.a.
Protein-bound FAs	4 & 5	OHFA	C32-C36	n.a	n.a	↓	n.a
Protein-bound lipids	3	Cer(OS)	C28-C36	↓	n.a.	varied	n.a.

Fractionated unbound and protein-bound epidermal lipids of newborn mice were analyzed by ESI-MS and quantified as described in Materials and Methods. Changes in lipid abundance in *Fatp4*^−/−^ mice compared to controls were summarized in 4 categories based on the chain length (≥C25 or <C25) and saturation (saturated or unsaturated) of the fatty acyl chains. 1-O-Cer(ENS) species that carry two saturated acyl chains are categorized as saturated whereas those that carry one or two unsaturated acyl chains are categorized as unsaturated. ^a^amide-linked acyl chain or acyl backbone; ^b^1-O-linked acyl chain; ^c^assessed by TLC; ^d^not for C16; ^e^not for all species; ↑ and ↓, abundance significantly increased or decreased, respectively, compared to controls; ≅, abundance unchanged compared to controls; ?, abundance unknown; n.a., non-applicable.

That *Fatp4*^−/−^ newborn epidermis revealed a switch to a less hydrophobic lipid composition is in agreement with the defective barrier that we previously reported^39^. The correlation between lipid composition and skin barrier is reminiscent of our previous findings that type II diester wax synthesized by sebaceous glands expressing a subnormal level of FATP4 bore fatty acyl moieties of a shorter carbon chain length with a higher degree of unsaturation. Those changes resulted in an increased fluidity of the sebum and less waterproofing of the fur^40^.

Data from several other animal models also support the importance of Cer(EOS) in permeability barrier function and perinatal survival, including mice deficient in ELOVL4^31,41–43^, ceramide synthase 3^44^, patatin-like phospholipase domain-containing 1 (PNPLA1)^45–48^, acyl CoA:diacylglycerol acyltransferase 2^49^, α/β hydrolase domain containing protein 5 (ABHD5)^36^, stearoyl CoA desaturase 2^50^, and sphingolipid activator protein precursor/prosaposin^26^. These proteins are all involved in pathways contributing to synthesis or availability of Cer(EOS). In contrast, animal models that display non-lethal phenotypes and relatively mild barrier defects do not show changed levels of epidermal Cer(EOS). For example, whereas *Fatp4*^−/−^ mice showed at least 200% increased transepidermal water loss vs. controls^14^, *Acbp*^−/−^ mice^51^ deficient in acyl-CoA binding protein and *Elovl3*^−/−^ mice^52^ lacking the enzyme in elongating fatty acyl chains up to 24 carbons^53^ showed only ~50% and ~70% increased transepidermal water loss, respectively.

The identification of Cer(BS) and MAG in this study and of Cer(BS) in our recent report on human stratum corneum lipids^27^ is unique. To our knowledge, Cer(BS) and MAG have not been identified in human or other mammalian skin. *Fatp4*^−/−^ epidermis showed dramatically decreased proportions of all MAG species carrying a saturated acyl chain ranging from 22 to 30 carbons. In an *in vitro* study, a mixture of MAG species containing a saturated acyl chain ranging from 22 to 26 carbons displayed better occlusive properties than petroleum jelly, which contains a mixture of hydrocarbons, suggesting a role for MAG in the skin barrier^54^. It is unlikely that the epidermal MAG was mainly produced from enzymatic or non-enzymatic lipolysis of triacylglycerols and diacylglycerols. First, our MS analyses revealed that epidermal triacylglycerols in control mice comprised only 46 to 56 carbons in total in the three fatty acyl chains esterified to the glycerol backbone (data not shown), with those carrying 52 and 54 carbons being the most abundant species, indicating shorter acyl chains in triacylglycerols than in MAG. Second, the reduced level of MAG in *Fatp4*^−/−^ epidermis was accompanied by an unaltered level of triacylglycerols, as shown in our previous study^14^, implicating no correlation in production between these two lipids. From where epidermal MAG is derived remains to be investigated.

With a stratum corneum lipid model, it has been reported that the absence of Cer(EOS) or substituting FFA with shorter chain length ones results in altered lipid lamellae organization and increased permeability^55–57^. Effects of perturbed lipid composition on intercellular lipid lamellae organization and/or barrier function have been revealed in human skin disorders such as atopic eczema and Netherton syndrome^58–60^, two inflammatory diseases that manifest with skin barrier defects and allergy features. Some of the lipid composition defects shared by these disorders were also observed in *Fatp4*^−/−^ epidermis, including a reduced level of long-chain ceramides and increased levels of short-chain ceramides, Cer(AS), and monounsaturated FFA.

Changes in abundance of Cer(EOS) and several Cer(AS) ≥ C22 species in mutants were well reflected in their corresponding GlcCer precursors, but changes in Cer(NS), Cer(NP) and Cer(AS)C16 were not. This suggests that GlcCer is not the only precursor for these ceramides, consistent with the finding in mouse and human epidermal lipids that ceramides 2 (Cer(NS)) and 5 (Cer(AS)C16) could also be derived from sphingomyelin packaged in the lamellar bodies^61^.

Our data showed that the bound Cer(OS) in *Fatp4*^−/−^ epidermis was significantly reduced and switched from a more to a less hydrophobic composition. Our recent data from patients and animal models with mutations in epidermal lipid synthesis indicate that the corneocyte lipid envelope originates from the limiting membrane of lamellar bodies and functions as a bidirectional scaffold for the formation of both intercellular lipid lamellae and cornified envelope^25^. Thus the abnormalities in bound Cer(OS) likely contribute to the defective barrier in *Fatp4*^−/−^ mice. Several other animal models showing defective skin barrier also displayed alterations in ultrastructure of corneocyte lipid envelope or depletion or reduction in bound Cer(OS) levels^26,36,41,44,45,62,63^. In 12R-lipoxygenase- and ABHD5-deficient epidermis, both saturated and unsaturated bound Cer(OS) species were dramatically reduced. In contrast, FATP4-deficient epidermis showed depletion in all saturated bound Cer(OS) species but various changes in unsaturated Cer(OS) species.

With the substrate preference of FATP4 towards saturated NHFA and BHFA (≥C25) and saturated or unsaturated OHFA (≥C30), it is expected that these FFA molecules would accumulate inside *Fatp4* mutant keratinocytes due to an inability of cells to drive the formation and usage of their acyl-CoA derivatives. However, by MS the FFA that showed the most dramatic elevation in abundance in *Fatp4*^−/−^ epidermis was unsaturated NHFA (≥C26). It is possible that this resulted from conversion of saturated NHFAs into unsaturated ones to prevent lipotoxicity (see below). Alternatively, the accumulation of FFA in *Fatp4*^−/−^ epidermis could also result from faster delivery of FAs into cells by an unknown mechanism, increased synthesis of FAs, or increased degradation of other lipids into FFA. Accumulation of FFA in the skin has also been reported in other animal models. For example, a similar compensatory response to impaired Cer(EOS) synthesis and skin barrier was observed in PNPLA1-deficient mice^46^.

The observed abnormalities in FFA could change the stratum corneum pH or cause cytotoxicity^6^. For example, the decreased level of FFA in *Acbp*^−/−^ mice is accompanied by an increased pH in the stratum corneum, which may affect the ionization of FAs, leading to instability of the lamellar membrane and a defective barrier^51^. In cultured cells it was reported that saturated LCFA like palmitic acid leads to cell death through lipid remodeling, oxidative stress, or endoplasmic reticulum stress, whereas unsaturated FFA like oleic acid protects from lipotoxicity^64,65^. Lipotoxicity can also result from the detergent effect of excess FFA with calcium^66^.

In summary, we obtained epidermal lipid data by high resolution MS for accurate results, verified the presence of epidermal Cer(BS) and MAG by MS, and verified the lipid band of Cer(NP) on TLC plates. Our lipidomic analysis also represents the most thorough such study of the permeability barrier in *Fatp4*^−/−^ mice to date, providing information about how FATP4 can contribute to barrier function by regulating fatty acyl moieties in various barrier lipids including ceramides and MAG. Understanding how FATP4 regulates lipid metabolism is important for elucidating the pathogenesis and potential therapies for ichthyosis prematurity syndrome and related disorders.

## Materials and Methods

### Mice

*Fatp4* mutant (*Slc27a4*^*wrfr*^) and human involucrin promoter-driven Fatp4 and Fatp1 transgenic mice have been previously described^14^. All animal experiments conformed to the NIH Guide for the Care and Use of Laboratory Animals and were approved by the Washington University Institutional Animal Care and Use Committee.

### Electron microscopy

The ultrastructural analysis by EM was performed as described^25^. To visualize the corneocyte lipid envelope and quantify cornified envelope dimensions, skin samples were flash frozen and thawed in absolute pyridine for 2 h at RT. Following standard fixation, samples were post-fixed in reduced osmium tetroxide or ruthenium tetroxide before epoxy embedding. The samples were cut on a Leica Ultracut E microtome (Leica microsystems, Wetzlar, Germany) and imaged on a JEOL 100CX transmission electron microscope (JEOL, Tokyo, Japan) using a Gatan digital camera. For quantification, the thickness of the cornified envelope was measured in at least 25 randomly selected positions in 5 random high-powered electron micrographs of the mid stratum corneum from two mice of each genotype. The observer recording these measurements was blinded to the groups.

### Isolation of cornified envelope

Dorsal skin (5 × 5 mm) of embryonic day 16.5 or older mouse embryos was minced using a razor blade and boiled for 5 min in 200 µl of extraction buffer containing 0.1 M Tris, pH8.5, 2% SDS, 20 mM DTT, 5 mM EDTA, pH8.0. After 10 min of centrifugation, the pellet was resuspended in 100 µl of extraction buffer, put onto slides, and photographed in phase contrast under a Nikon Eclipse E800 microscope^67^.

### Extraction of epidermal lipids

To obtain free extractable lipids, a lyophilized 10 mg epidermis sample prepared as described^14^ was soaked in 0.8 ml water in a centrifuge tube for 5 min, and 3 ml chloroform/methanol (1:2, v:v) were added. Following 30 sec of vortexing and 2 h of shaking at room temperature, an additional 1 ml chloroform and 1 ml water were added. The extraction tube was vortexed for another 30 sec, centrifuged at 2,000 rpm for 5 min, and the lower, organic layer and the epidermis was each transferred to new tubes. The organic layer was re-extracted in 1 ml chloroform, 1 ml methanol, and 0.9 ml water with vortexing and centrifugation. The organic phase was transferred to a vial, dried with a nitrogen evaporator (Organomation Associates, Inc., Berlin, MA), resuspended in 200 µl chloroform/methanol (2:1), and stored at −20 °C.

To extract protein-bound lipids, the epidermis obtained from the extraction above was depleted of residual free lipids by shaking for 1 h sequentially in 2 ml chloroform/methanol mix at a 1:1, 1:2, and 0:2 ratio. After discarding the extract, 1 ml 50 mM NaOH in methanol was added to the epidermis followed by incubation at 56 °C for 2 h with occasional mixing. The reaction was then neutralized with 30 µl of 2 N HCl and extracted in 2 ml chloroform and 2 ml water. The organic layer was collected in a vial. The upper phase and the epidermis were reextracted with 2 ml chloroform, and the organic phase was collected and pooled with the previous extract. The pooled extract was dried, resuspended, and stored as described above.

For quantification of free extractable lipids by MS, lipid standards were added to samples at the beginning of extraction as follows: 1 µg 1-oleoyl-N-heptadecanoyl-D-*erythro*-sphingosine (18:1-d18:1/17:0-Cer(1-O-ENS); 860526 from Avanti Polar Lipids, Alabaster, AL) for Cer(1-O-ENS); 3 µg tridecanoic acid (13:0-FA; 1161 from Matreya LLC, Pleasant Gap, PA) for FAs; 2 µg N-decanoyl-D-*erythro*-sphingosine (d18:1/10:0-Cer(NS); 1333 from Matreya) for Cer(NS), Cer(NdS), and Cer(EOS); 11 µg monoheptadecanoin (17:0-MAG; M-159 from Nu-Chek Prep, Elysian, MN) for MAG; 1 µg N-alpha-hydroxydodecanoyl-D-*erythro*-sphingosine (d18:1/αh12:0-Cer(AS); 2042 from Matreya) for Cer(AS), Cer(AdS), Cer(BS), Cer(OS), and Cer(NP); and 0.2 µg D-glucosyl-β1-1’-N-dodecanoyl-D-*erythro*-sphingosine (d18:1/12:0-GlcCer(NS); 860543 P from Avanti) for all GlcCer.

For quantification of protein-bound lipids by MS, lipid standards were added to samples at the beginning of incubation in alkaline solution as the following: 1 µg 17-hydroxyheptadecanoic acid (ωh17:0-OHFA; 1760 from Matreya) for OHFA; and 1 µg N-alpha-hydroxydodecanoyl-D-*erythro*-sphingosine for Cer(OS).

### Lipid analysis and quantification by TLC

Flexible silica gel 60 plates (Sigma, St. Louis, MO) were baked at 100 °C for 30 min in an oven prior to use. Free extractable or protein-bound lipids, along with lipid standards, were applied to plates and resolved in a solvent mixture of chloroform/methanol/water (40:10:1) in a pre-equilibrated tank until the solvent front moved 7 cm from the bottom of the plate. The plates were air-dried for 5 min and re-resolved twice in a mixture of chloroform/methanol/glacial acetic acid (95:4.5:0.5) until the solvent front reached the top of the plate, with 5-min air-dry between the two developments. Resolved plates were dried, and lipid spots were visualized by spraying with 3% copper acetate in 8% phosphoric acid followed by charring at 180 °C for 15 min^68^.

For quantification of lipid types, lipid spots on charred TLC plates were scanned and quantified in ImageJ (http://rsbweb.nih.gov/ij/) with co-chromatographed standards as follows: Oleic acid (18:1-FA; U-46A from Nu-Chek) in the range of 0.5 to 4 µg for quantifying OAHFA, and in the range of 0.5 to 8 µg for OHFA; monoolein (18:1-MAG; 1787-1AMP from Sigma) in the range of 0.5 to 4 µg for MAG; bovine ceramides with NHFA (1056 from Matreya) in the range of 0.265 to 2.12 µg for Cer(1-O-ENS), Cer(EOS), and Cer(NS); bovine ceramides with AHFA (1056 from Matreya) in the range of 0.235 to 1.88 µg for Cer(AS), Cer(BS), Cer(NP), and lipid X, and in the range of 0.235 to 3.76 µg for Cer(OS); and bovine galactosylceramides (1128 from Matreya) in the range of 0.5 to 4 µg for all GlcCer bands. The amount of each lipid band was indicated in µg/mg of dry epidermal weight.

### Fractionation of lipids

Fractionation of lipids was performed as previously described with some modifications^69^. Crude lipids were dried, re-dissolved in 500 µl chloroform, and loaded onto a 3 mL/500 mg Macherey-Nagel (Duren, Germany) amino Chromabond Sep-Pak column that was pre-washed with 2 × 3 mL hexane. The column was first eluted with 3 mL hexane:diethyl ether (90:10) (Fraction 1), followed by 3 mL hexane:ethyl acetate (75:25) (Fraction 2), 3 mL chloroform:methanol (15:1) (Fraction 3), 2 × 3 mL diisopropyl ether:acetic acid (98:5) (Fractions 4 and 5), 3 mL acetone:methanol (9:1.4) (Fraction 6), 3 mL chloroform:methanol (2:1) (Fraction 7), and finally eluted with 3 mL chloroform:methanol (1:2) (Fraction 8) by gravity. The eluants were dried in nitrogen, re-dissolved in chloroform (for fractions 1 to 5) or chloroform/methanol (2:1) (for fractions 6 to 8), and stored at −20 °C.

### Recovery of lipids from TLC plates

To recover lipid bands from TLC pates, crude or fractionated lipids were resolved on TLC plates that had been blank-developed in chloroform/methanol (1:1) overnight to remove impurities. The resolution condition was as described above except that the preparative plate of fraction 2 lipids was resolved in a mixture of chloroform/methanol/glacial acetic acid (95:4.5:0.5) until the solvent front moved 16 cm from the bottom of the plate and then in a mixture of hexane/diethyl ether/acetic acid (75:25:1) until the solvent front reached the top of the plate. One of the resolved sample lanes was cut out from the plate and charred as fiduciary markers. With alignment to the charred lane, lipid bands of interest on the uncharred plate were marked with a pencil, scraped out with a razor blade, and extracted by vortexing in chloroform:methanol (2:1; 1.5 ml/cm^2^ gel scraped) for 30 sec followed by centrifugation. The extract was then collected, dried, resuspended in chloroform or chloroform/methanol (2:1), and stored at −20 °C.

### Lipid analysis and quantification by MS

Lipid analysis was performed on a Thermo Scientific (San Jose, CA) LTQ Orbitrap Velos mass spectrometer with Xcalibur operating system as described previously^27^. Fractionated lipids or lipids recovered from TLC plates were diluted in methanol with 1% NH_4_OH and loop injected into the ESI source using a built-in syringe pump that delivered a constant flow of methanol with 1% NH_4_OH at a flow rate of 15 µl/min. The ESI needle was set at 4.0 kV, and temperature of the heated capillary was 300 °C. The automatic gain control of the ion trap was set to 5 × 10^4^, with a maximum injection time of 50 ms. Helium was used as the buffer and collision gas at a pressure of 1 × 10^−3^ mbar (0.75 mTorr). The MS^n^ (n = 2, 3) spectra were acquired for structural identification^27^, and the experiments were carried out with an optimized relative collision energy ranging from 30–45% and with an activation q value at 0.25, and an activation time at 10 ms to leave a minimal residual abundance of precursor ion (around 20%). The mass selection window for the precursor ions was set at 1 Da wide to admit the monoisotopic ion to the ion-trap for CID for unit resolution detection in the ion-trap or high-resolution accurate mass detection in the Orbitrap mass analyzer. Mass spectra were accumulated in the profile mode, typically for 2–10 min for MS^n^ spectra (n = 2–4). Lipid quantitation was achieved by high resolution ESI-MS measurement on the lipid fractions with internal standards that were added prior to extraction. All ceramides were analyzed as the [M − H]^−^ ions in the negative-ion mode except for 1-O-acylceramides which were analyzed as the [M + H]^+^ ions in the positive-ion mode. MAG was detected as the [M + NH_4_]^+^ ions in the positive ion mode. The intensity ratio of individual lipid species to the internal standard was calculated, normalized to the dry epidermal weight, and the abundance of individual lipid species was plotted in arbitrary units.

### Nomenclature

The following designations and abbreviations recommended by IUPAC (https://www.qmul.ac.uk/sbcs/iupac/lipid/) are used. The designation of ceramide is in the form of d(or t) long-chain base /FA, with d denoting a dihydroxy and t denoting a trihydroxy long-chain base. Thus, for example, the sphingosine (sphing-4-enine) and sphinganine long-chain bases are designated as d18:1, and d18:0, respectively. Fatty acyl moieties with or without hydroxyl substituent are denoted as hFA or nFA, respectively, and sphingosine ceramides with α-, β-, or ω-hydroxyl fatty acyl substituent are designated as d18:1/αhFA-Cer, d18:1/βhFA-Cer, d18:1/ωhFA-Cer, respectively. Therefore, N-α-, N-β-, and N-ω-hydroxy-palmitoyl-sphingosine, for example, are designated as d18:1/αh16:0-Cer, d18:1/βh16:0-Cer, d18:1/ωh16:0-Cer, respectively. To categorize the ceramide subfamilies, the nomenclature of Motta *et al*.^70^, expanded by Robson *et al*.^71^ and Masukawa *et al*.^72^, was adopted. The initial letter of the sphingoid bases S, dS, P, and H are used to represent sphingosine, dihydrosphingosine, phytosphingosine, and 6-hydroxysphingosine, respectively, and the FA residues N, A, B, and O represent nonhydroxylated acyl, α-hydroxyacyl, β-hydroxyacyl, and ω-hydroxyacyl, respectively. Thus, for example, the d18:0/16:0-Cer, d18:1/βh16:0-Cer, and d18:1/ωh16:0-Cer belong to the Cer(NdS), Cer(BS), and Cer(OS) subfamilies, respectively.

### Statistical analysis

Two-tailed, unpaired Student’s *t*-tests were used to determine statistical differences in cornified envelope measurements. Two-way mixed model ANOVA tests were used in quantification of lipids by TLC and ESI-MS. Differences were considered significant when *P* < 0.05.

## Supplementary information

Supplementary Information

## Data Availability

Lipidomics data have been deposited into the EMBL-EBI MetaboLights database^73^ with the identifier MTBLS1138. The dataset can be accessed at https://www.ebi.ac.uk/metabolights/MTBLS1138.
