# Supplementary material for: Fatty acid transport protein 4 is required for incorporation of saturated ultralong-chain fatty acids into epidermal ceramides and monoacylglycerols
Source: Sci Rep. 2019 Sep 13;9:13254. doi: 10.1038/s41598-019-49684-y (PMC6744566; doi:10.1038/s41598-019-49684-y)
Supplement: Supplementary file 1 — Supplementary Information [file 41598_2019_49684_MOESM1_ESM.pdf]

Supplementary Information

Fatty acid transport protein 4 is required for incorporation of saturated ultralong-chain fatty acids into epidermal ceramides and monoacylglycerols

Meei-Hua Lin, Fong-Fu Hsu, Debra Crumrine, Jason Meyer, Peter M. Elias, and Jeffrey H. Miner

Supplementary Figures S1-S10

The following Figures (S1-S10) are examples (not to be comprehensive) that illustrate structural identification of the epidermal lipids by high resolution and MS^n (n=1-4) mass spectrometry in the present study.

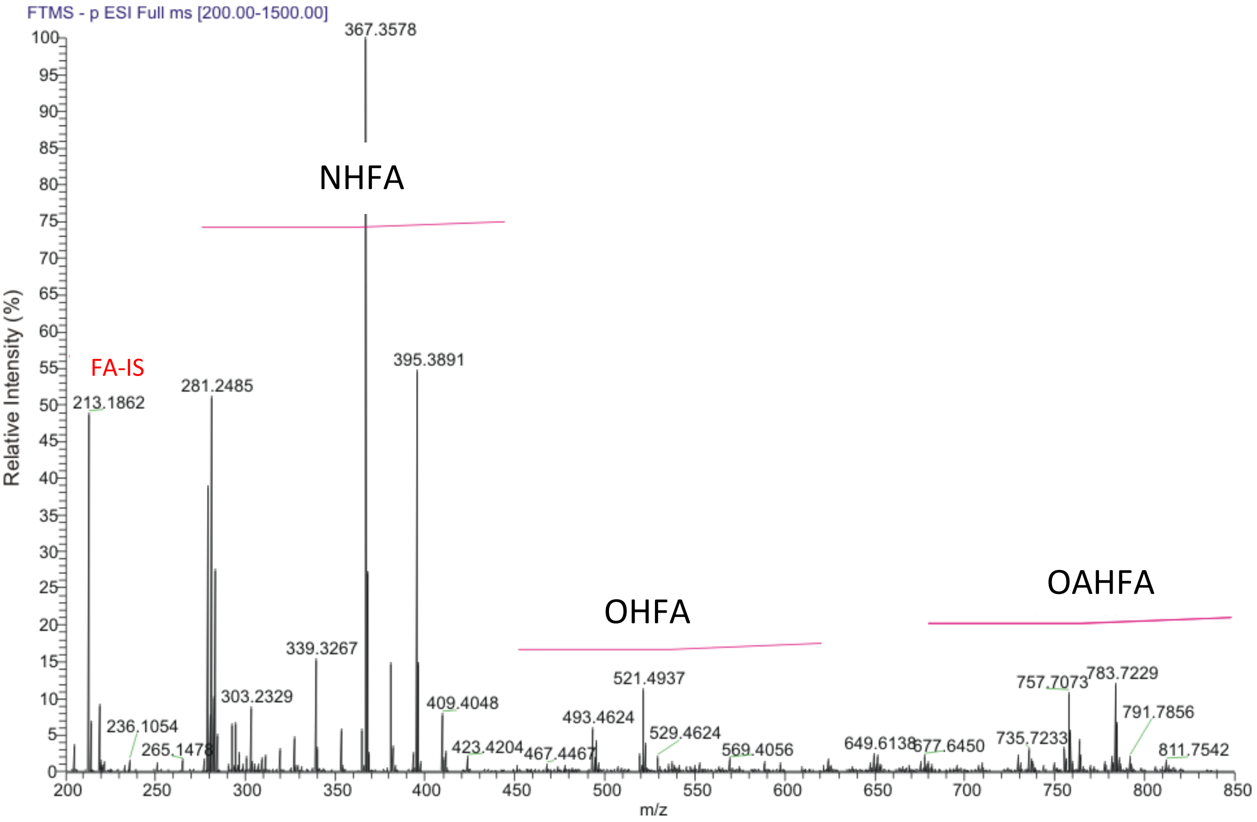

Fig. S1. The high resolution (R=100,000 at m/z 400) ESI-MS spectrum of the [M – H]- ions of unbound fatty acids (pooled fractions 4 and 5) from a newborn control mouse. Abundant fatty acids seen are NHFA, OHFA, and OAHFA. The ion at m/z 213 is 13:0-FA internal standard (IS) added before extraction. Characterization of ions at m/z 521 (ωh34:1-FA) and 783 (ωh34:1-18:2-FA) are shown in Fig. S4 and Fig. S5, respectively.

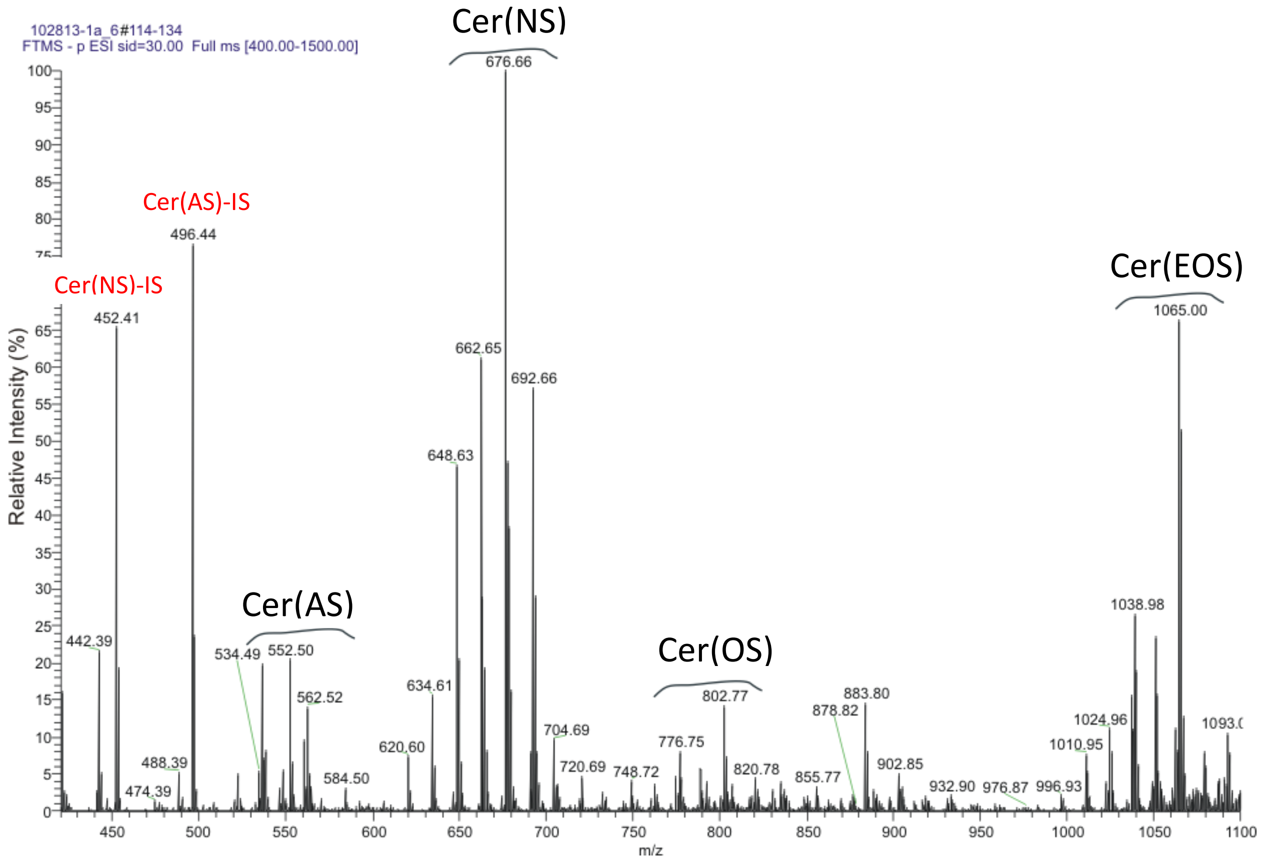

Fig. S2. The high resolution (R=100,000 at m/z 400) ESI-MS spectrum of the $[M-H]^-$ ions of fraction 3 of unbound epidermal lipids from a newborn control mouse. Abundant lipid families seen are Cer(AS), Cer(NS), Cer(OS) and Cer(EOS). The ions at m/z 452 (d18:1/10:0-Cer(NS)) and 496 (d18:1/αh12:0-Cer(AS)) are internal standards (IS) added before lipid extraction. Further MS^2 on these $[M-H]^-$ species using linear ion trap (LIT) is readily applicable for their structural characterization. The LIT MS^2 method is exemplified by identification of the Ion at m/z 802.77 (d18:1/ωh34:1-Cer(OS)), which is depicted in Fig. S9.

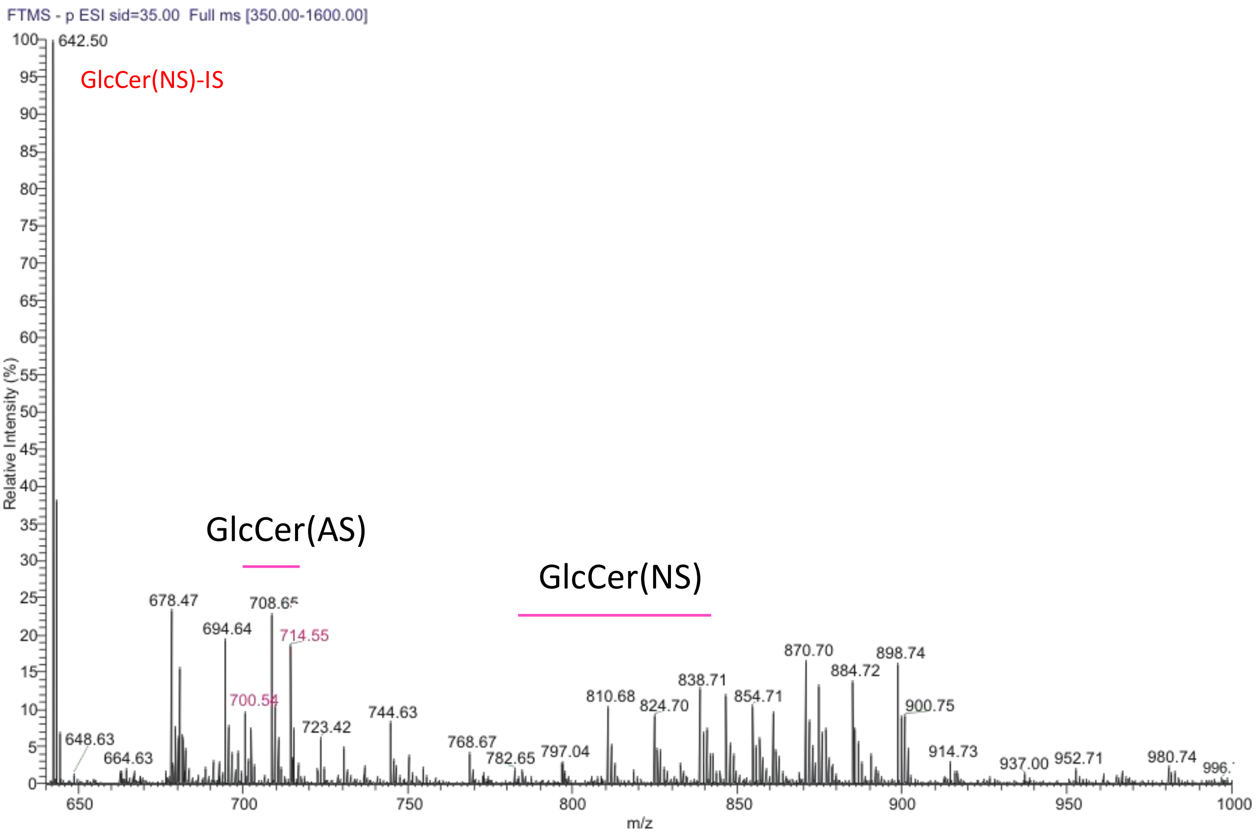

Fig. S3. The high resolution (R=100,000 at m/z 400) ESI-MS spectrum of the $[M - H]^-$ ions of fraction 6 of unbound epidermal lipids from a newborn control mouse. Abundant lipid families seen are GlcCer(AS) and GlcCer(NS). The ion at m/z 642 is d18:1/12:0-GlcCer(NS) internal standard (IS) added before lipid extraction.

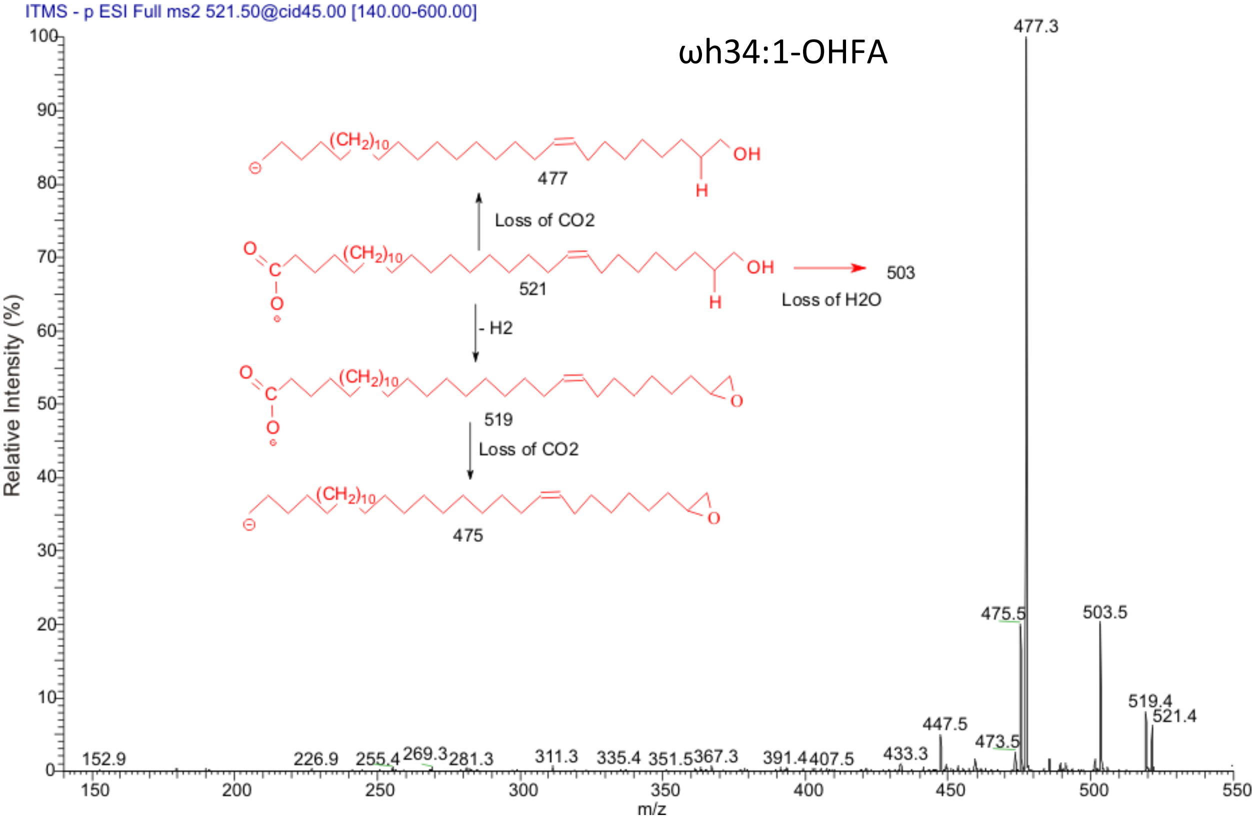

Fig. S4. The MS[2] spectrum of the [M – H]- ion of the ωh34:1-OHFA at m/z 521 of unbound epidermal lipids from a newborn control mouse, which undergoes traditional losses of H_2O (m/z 503) and CO_2 (m/z 477). The observation of the ions of m/z 519, may arise from loss of H_2 involving the hydrogen of the terminal OH group, forming a terminal epoxy fatty acid, which undergoes loss of CO_2 to form ions of m/z 475. The loss of H_2 possibly involving the participation of the hydrogen of the terminal OH may indicate the presence of the ω-OH group.

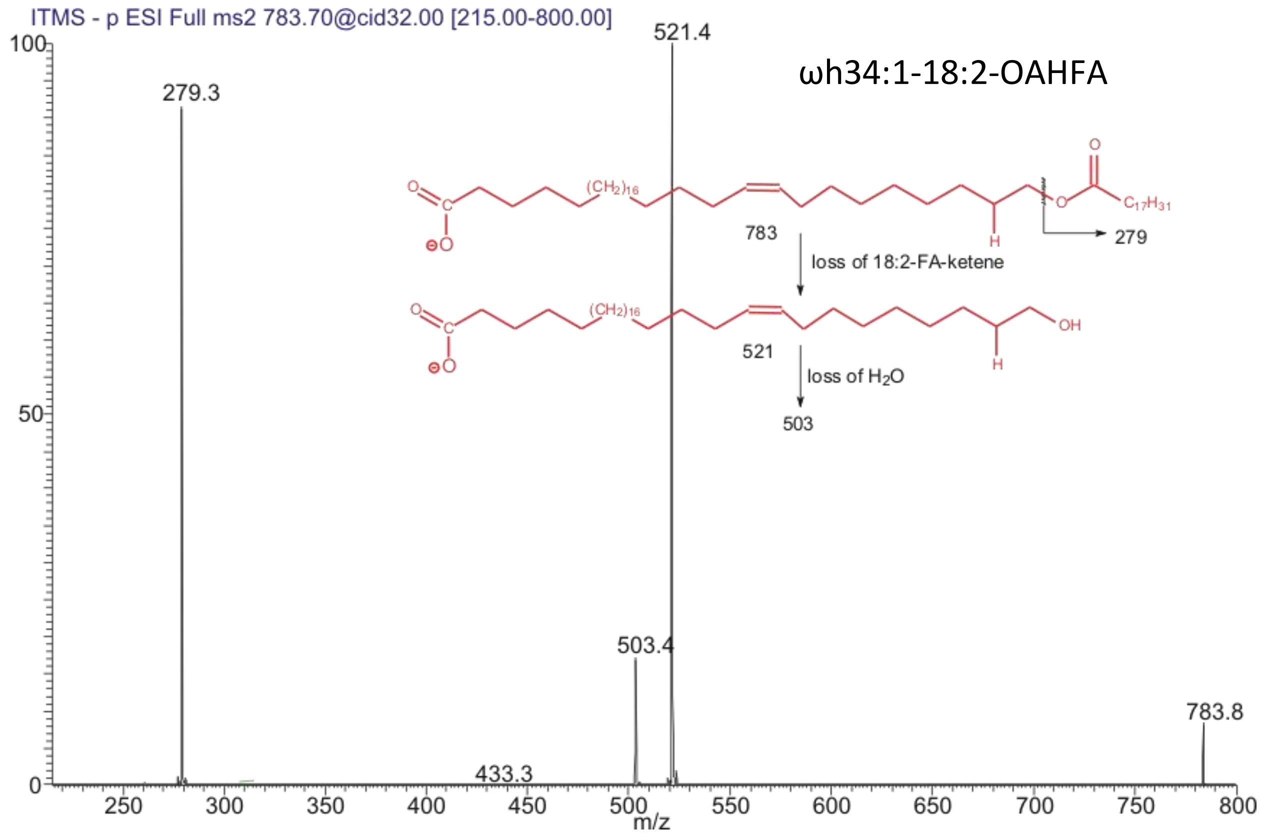

Fig. S5. The MS[2] spectrum of the [M – H]- ion of the ωh34:1-18:2-OAHFA at m/z 783 of unbound epidermal lipids from a newborn control mouse. The fragmentation pathways leading to structural identification are shown in inset.

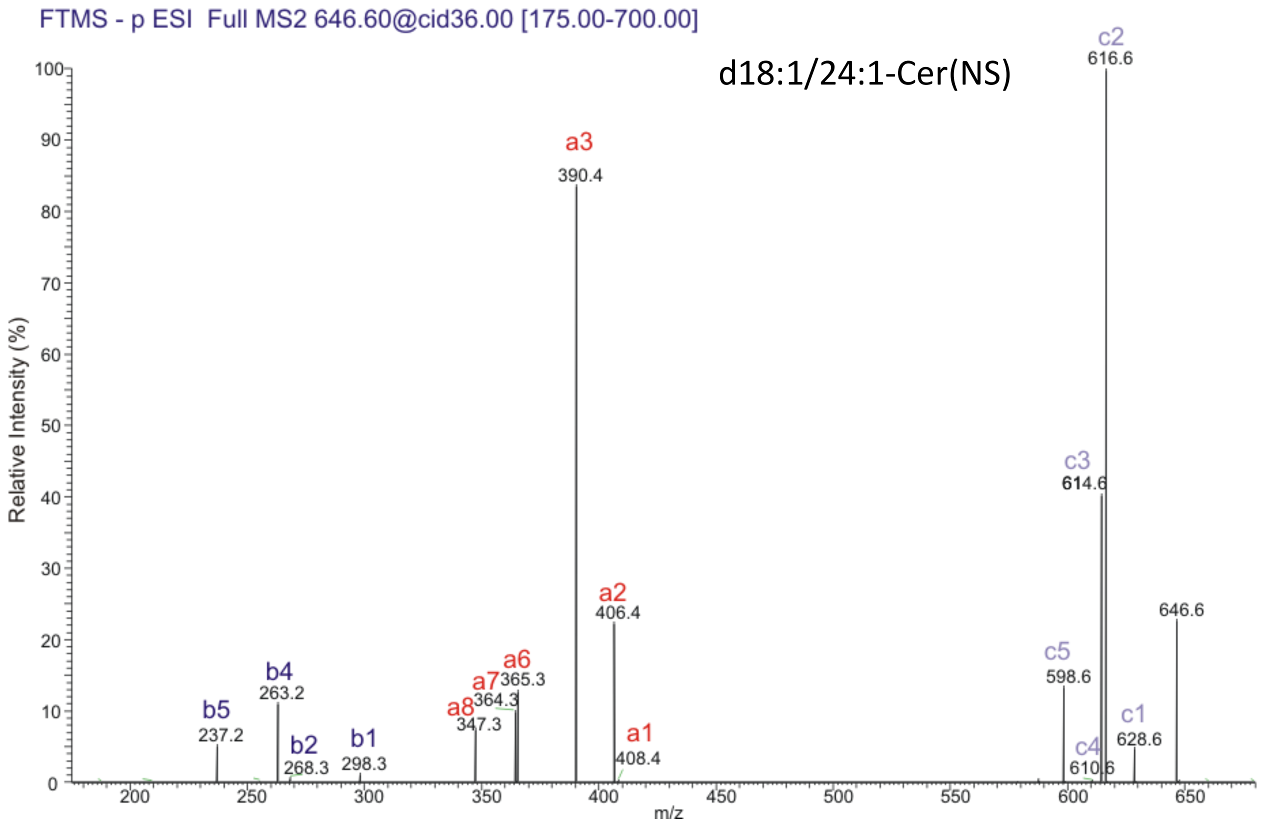

Fig. S6. The MS^2 spectrum of the [M – H]- ion of the d18:1/24:1-Cer(NS) at m/z 646 of unbound epidermal lipids from a newborn control mouse, in which ions from c-series (common ions for ceramides), a-series (ions related to fatty acyl chains), and b-series (ions to identify the long-chain base) are shown. Ion designations were adopted from reference 27. The presence of the multiple ion species in all the three ion series readily gave confident assignment of the Cer(NS) structure.

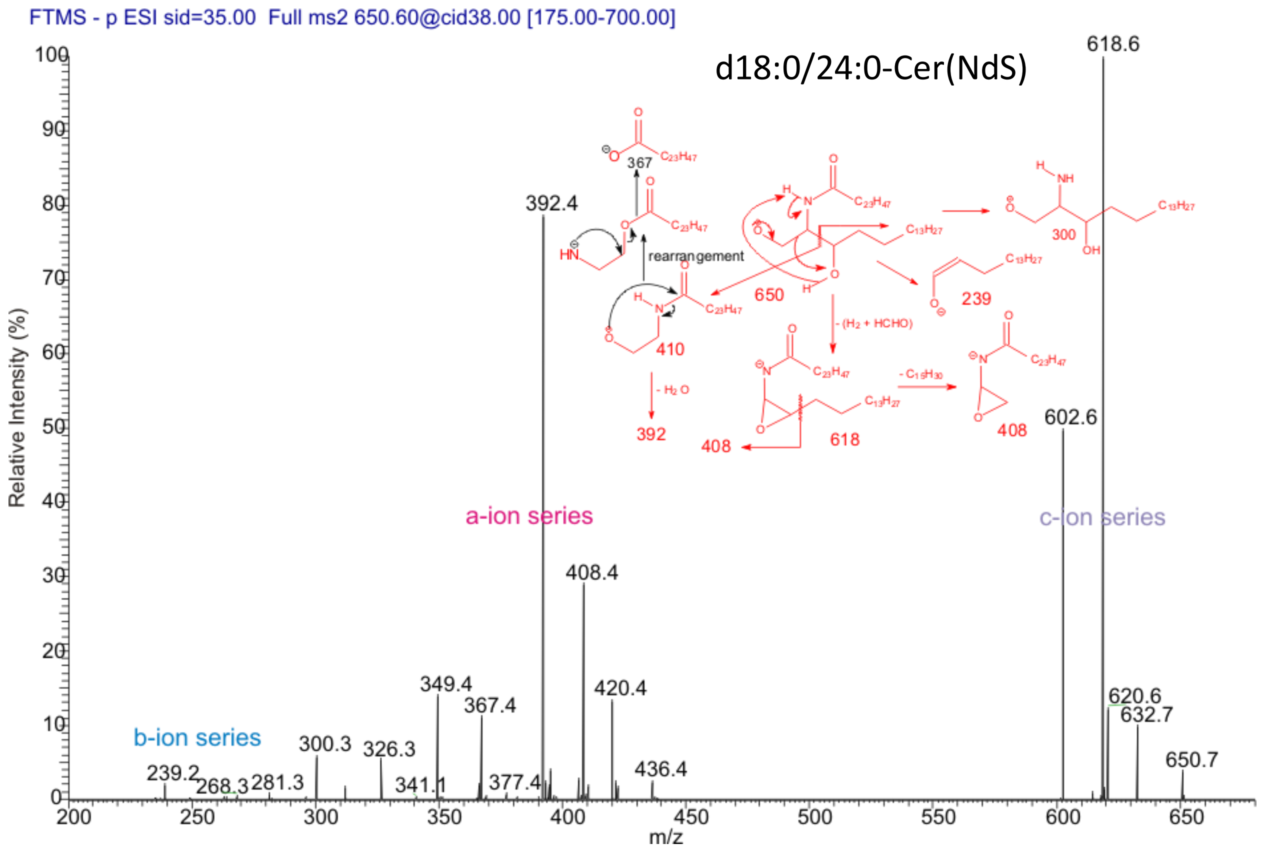

Fig. S7. The MS[2] spectrum of the [M – H]- ion of the d18:0/24:0-Cer(NdS) at m/z 650 of unbound epidermal lipids from a newborn control mouse, in which all the c-, a- and b-ion series are abundant and readily recognizable (not specifically marked). The proposed fragmentation pathways leading to the formation of the major fragment ions in the a- and b-ion series are shown in the inset, which illustrate how the structure can be identified.

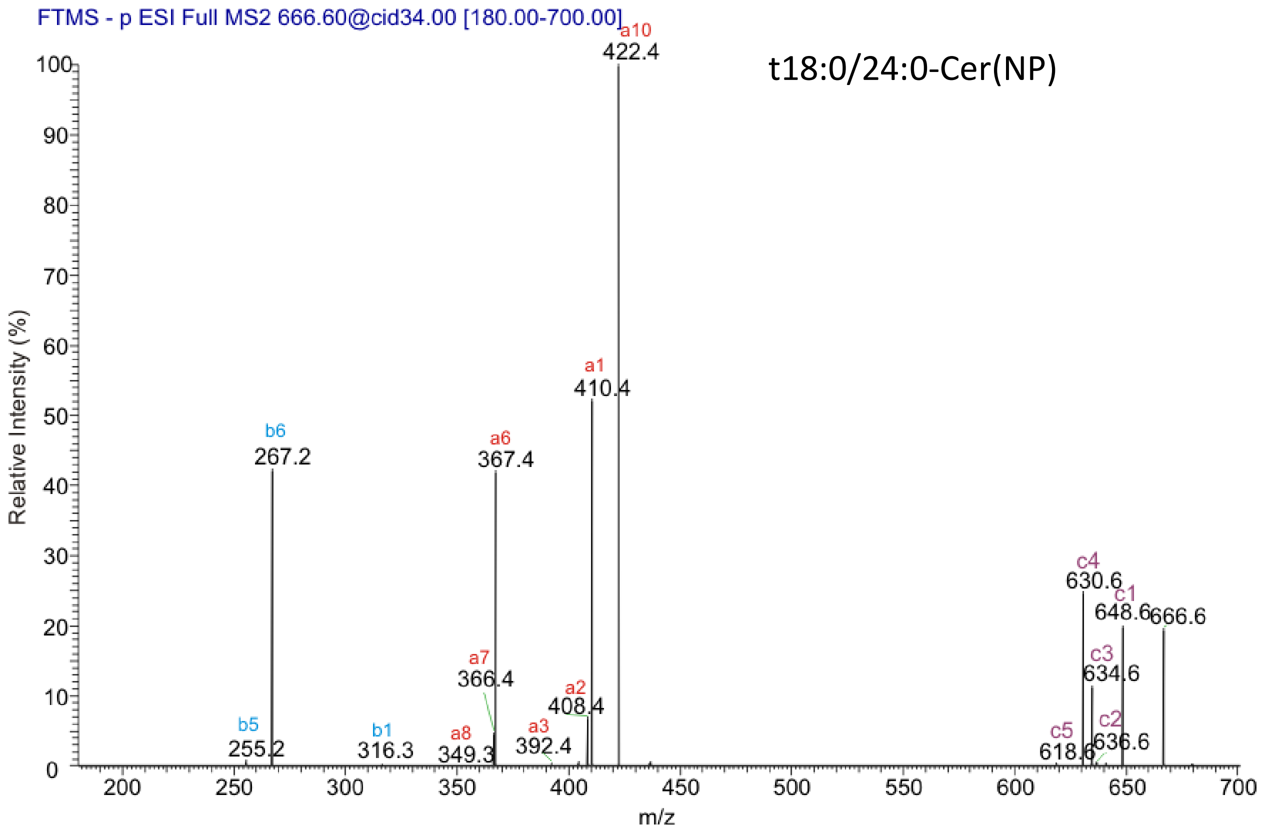

Fig. S8. The MS^2 spectrum of the $[M - H]^-$ ion of the t18:0/24:0-Cer(NP) at m/z 666 of unbound epidermal lipids from a newborn *Fatp4*$^{-/-}$ mouse, which represents a typical MS^2 spectrum from a Cer(NP) family, where ions from c-series, a-series, and b-series are unique and readily gave structural assignment of the t18:0/24:0-Cer(NP) structure.

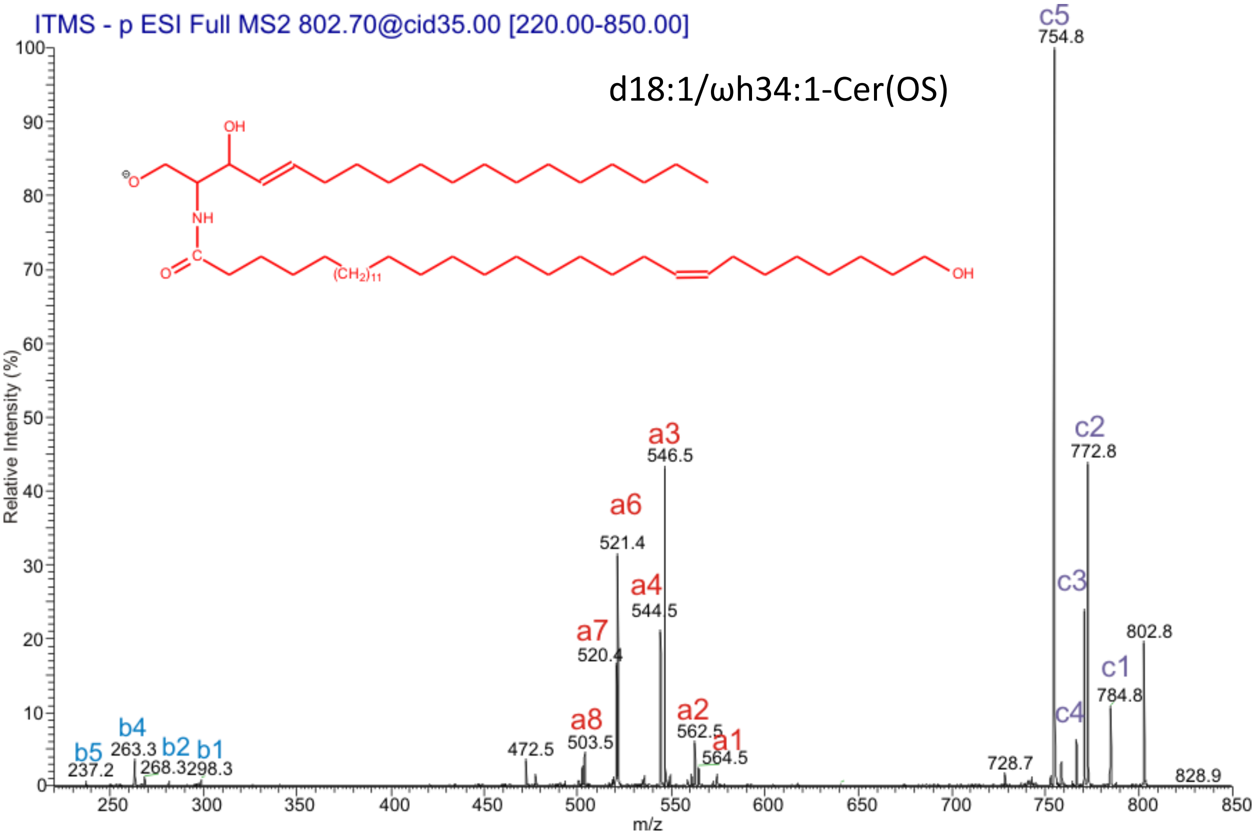

Fig. S9. The MS[2] spectrum of the [M – H]- ion of the d18:1/ωh34:1-Cer(OS) at m/z 802 of unbound epidermal lipids from a newborn control mouse, in which ions designated as c-series (common ions for ceramides), a-series (ions related to fatty acyl chains), and b-series (ions to identify the long-chain base) are shown. Ion designations were adopted from reference 27. These characteristic ions for Cer(OS) readily lead to structural identification.

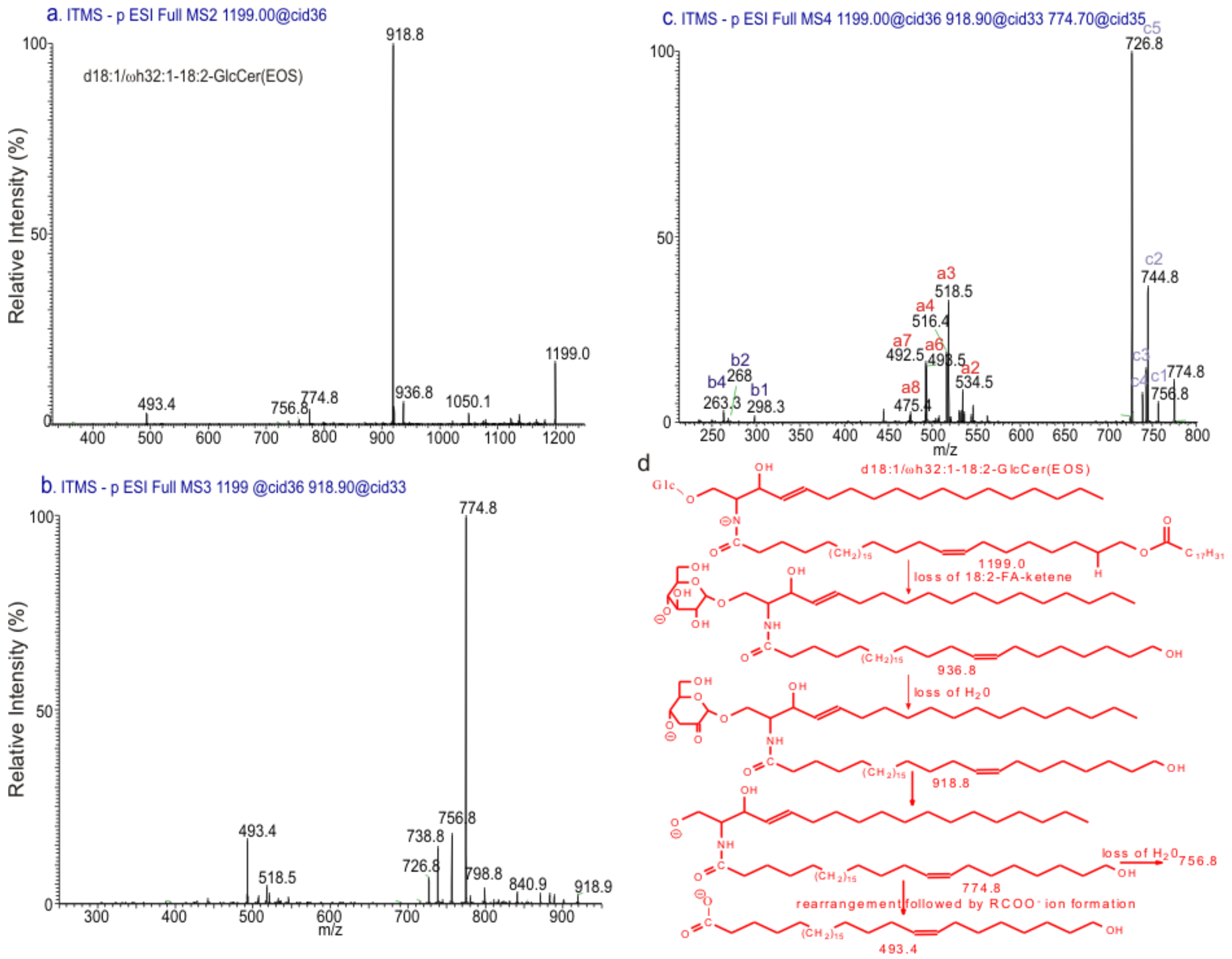

Fig. S10. The MS^2 spectrum of the [M – H]- ion of the d18:1/ωh32:1-18:2-GlcCer(EOS) at m/z 1199 (a), its MS^3 spectrum of the ion of m/z 918 (1199 → 918) (b), and MS^4 spectrum of the ion of m/z 774 (1199 →918 → 774) (c) of unbound epidermal lipids from a newborn control mouse. Panel (d) shows the proposed fragmentation that leads to the identification of the structure. Because of the structural complexity, multiple stage mass spectrometry (MS^n) is required to elucidate the structure, including the glucosyl (Glc) head group, 18:2-FA tail, and the Cer(OS) backbone. Panel (c) contains the c-, a-, and b-series ions that confirm the Cer(OS) core structure.
